# Afzelin induces immunogenic cell death against lung cancer by targeting NQO2

**DOI:** 10.1186/s12906-023-04221-3

**Published:** 2023-10-27

**Authors:** Lei Xia, Xiaoqing Xu, Meijun Li, Xinyue Zhang, Fang Cao

**Affiliations:** 1 Department of Medical Oncology, Affiliated Hospital of Shandong University of Traditional Chinese Medicine, No. 16369, Jingshi Road, Jinan, Shandong 250000 China; 2 The First Clinical College, Shandong University of Traditional Chinese Medicine, Jinan, 250355 Shandong China; 3 Department of Traditional Chinese Medicine, Zibo Central Hospital, Zibo, 255036 Shandong China

**Keywords:** Afzelin, Lung cancer, Immunogenic cell death, NQO2, Endoplasmic reticulum stress

## Abstract

**Background:**

Lung cancer is one of the most common malignant cancers worldwide. Previous studies have shown that Afzelin, a flavonoid, possesses anticancer activity. The aim of this study was to explore Afzelin’s effect on lung cancer cells and delineate potential anti-cancer mechanism.

**Methods:**

The effect of Afzelin on cell viability, proliferation, and apoptosis of lung cancer cells i.e., A549 and H1299 cells, was studied. The targets for Afzelin in lung cancer were predicted using SwissTargetPrediction, Next, the GO analysis and pathway enrichment were analyzed using String. For in vitro studies, the overexpression plasmid of NQO2, the identified target of Afzelin, was transfected into Afzelin-treated cells to verify the regulatory role of Afzelin on its target and signaling pathway.

**Results:**

In in vitro studies, Afzelin markedly inhibited cell viability, proliferation, and raised apoptotic rate of A549 and H1299 cells. In addition, Afzelin activated endoplasmic reticulum (ER) stress and increased ATP, HMGB1, and CRT levels in lung cancer cells, indicating that Afzelin induced immunogenic cell death (ICD). SwissTargetPrediction identified NQO2 as a target of Afzelin. Further, Afzelin markedly inhibited NQO2 protein expression and in turn, overexpression of NQO2 attenuated the effect of Afzelin on A549 and H1299 cells.

**Conclusion:**

Afzelin inhibits lung cancer progression by targeting NQO2, in turn, activating ER stress and inducing ICD.

**Supplementary Information:**

The online version contains supplementary material available at 10.1186/s12906-023-04221-3.

## Highlight

1. Afzelin suppresses lung cancer cell proliferation and accelerates their apoptosis

2. NQO2 is identified as a potential target for Afzelin in lung cancer cells

3. Afzelin induces endoplasmic reticulum stress and immunogenic cell death via NQO2

**Supplementary Information:**

The online version contains supplementary material available at 10.1186/s12906-023-04221-3.

## Background

Lung cancer is the most common cancer in the world and the leading cause of death due to cancer. Worldwide, there are more than 2.2 million new cases and 1.8 million deaths from lung cancer [1]. Pathologically, lung cancer is divided into small cell lung cancer (SCLC) and non-small cell lung cancer (NSCLC) [2]. Among lung cancer patients, NSCLC accounts for about 85% of all cases [3]. Five-year survival rate for advanced NSCLC patients is less than 20% due to difficulties in early diagnosis, drug resistance, and frequent recurrence [4]. Therefore, it is important to find effective drugs against lung cancer.

**Fig. 1:**
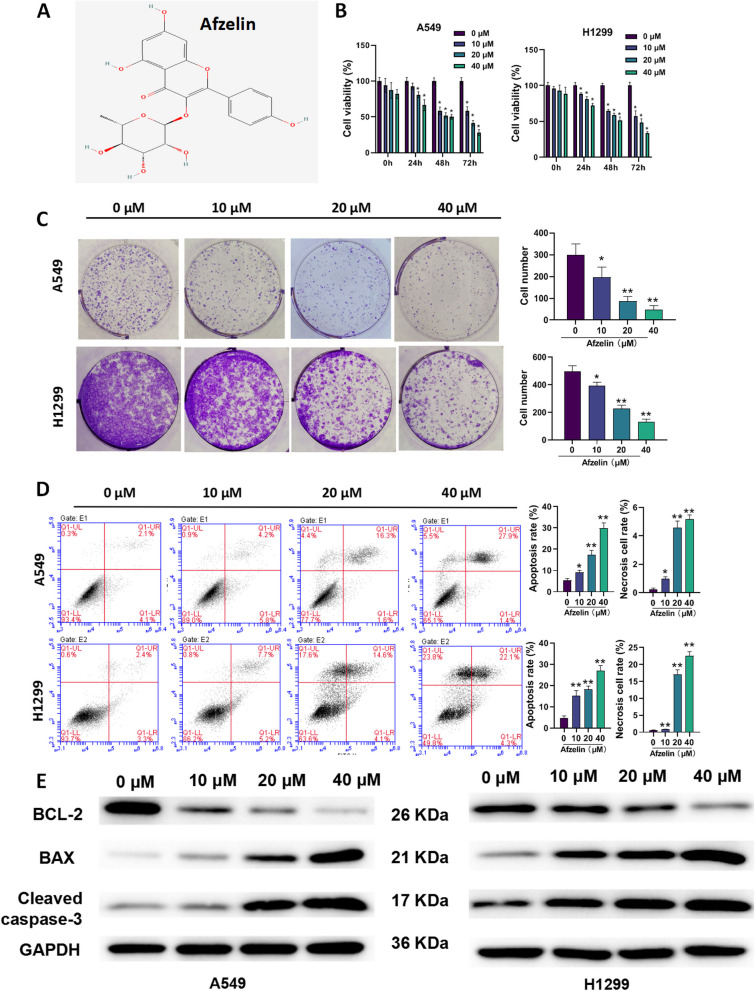
Afzelin inhibits proliferation and promotes apoptosis of lung cancer cells. **A** The chemical structure of Afzelin, **B** viability, **C** proliferation, **D** apoptosis, and **E** expression of Bax, Bcl-2, and cleaved caspase-3 in A549 and H1299 cells. *P < 0.05, **P < 0.01, ***P < 0.001

Afzelin (kaempferol 3-O-rhamnoside, Fig. 1A) is a flavonoid glycoside found in *Houttuynia cordata* Thunb [5]. In addition, Afzelin is found in a variety of edible plants such as annonoya, peppercorns, dried ginger, lotus, and *ginkgo biloba* [6]. The anti-inflammatory and antioxidant effects of Afzelin have been found useful in fulminant hepatic failure [7], ultraviolet B-induced skin damage [8], and cognitive function in Alzheimer’s disease [9]. More importantly, Afzelin inhibits breast cancer by stimulating apoptosis [10]. In addition, Afzelin has an anticancer effect on prostate cancer cells [11]. In particular, Afzelin was found to be cytotoxic to lung cancer cells, but the mechanism of cytotoxicity is yet to be established [12].

Immunogenic cell death (ICD) is a unique form of tumor cell death where the cells progress from non-immunogenicity to immunogenicity, triggering tumor immune effects in vivo and leading to cell death [13, 14]. ICD-induced dying cells release DAMPs, including ATP, HMGB1, calreticulin (CRT), and heat shock proteins (such as HSP70 and HSP90), which have been previously identified as specific molecular markers for ICD [15–17]. ICD can be triggered in response to various antitumor therapies, including targeted agents, radiation therapy, and chemotherapy [18]. Therefore, we hypothesized that Afzelin may inhibit the development of lung cancer via the induction of ICD. Several studies have investigated the effects of Afzelin on cellular processes and signaling pathways. For example, Jung et al. has shown that Afzelin positively regulates melanogenesis via the p38 MAPK pathway [19]. Jung E et al. also demonstrated that Afzelin protects melanocytes by activating the Nrf2- ARE pathway and inhibiting GSK-3β [20]. In addition, Shin et al. found that Afzelin exerts an antagonistic effect against ultraviolet B-induced cell damage [8]. In addition, Rachmi et al. identified potential targets of Afzelin in inhibiting the migration of triple-negative breast cancer using reverse docking [21]. Although these studies do not directly address ICD, they provide important information about the role of Afzelin in cellular processes and pathway regulation. Further studies could provide a more comprehensive understanding of the possible relationship between Afzelin and ICD.

Therefore, the primary objective of this study was to investigate the potential role of Afzelin in ICD and unveil its underlying molecular mechanisms involved in immune regulation. To achieve this, we conducted a series of experiments to examine the effects of Afzelin on the proliferation and apoptosis of lung cancer cells in vitro. Additionally, we utilized bioinformatic analysis to identify the targets of Afzelin and explored possible mechanisms associated with its action. The findings of our study present novel prospects for the development of promising therapeutic agents targeting lung cancer.

## Methods

### Cells and treatment

Lung cancer cell lines, A549 and H1299 (Procell), were cultured at 37 °C in RPMI-1640 medium supplemented with 10% FBS. Afzelin (MedChemExpress) was dissolved in ethanol and adjusted to 40, 20, 10, and 0 μM final concentration. Cells were treated with these different concentrations of Afzelin for 48 h at 37 °C.

### Cell transfection

The NQO2-overexpressing plasmid (NQO2) and the interfering plasmid (Vector) were transfected into cells using Lipofectamine 3000 (Invitrogen). After culturing for 24 h, cells were treated with 40 μM Afzelin and cultured for an additional 48 h. The overexpression plasmid and vector were obtained from GenePharma.

### CCK-8 assay

Cells were seeded in 96-well plates (5 × 10^3^ cells/well) and treated with various concentrations of Afzelin. CCK-8 solution (Beyotime) was added to each well and incubated for 2 h. Fluorescence was measured using a microplate reader at 450 nm.

### Colony formation assay

Proliferation of A549 and H1299 cells was determined using a colony formation assay. Cells were treated with Afzelin for 48 h or transfected with NQO2 and then seeded in 6-well plates (10^3^ cells/well). After 10–14 days, cells were fixed with 4% paraformaldehyde and stained with 0.1% crystal violet. Colony number was calculated using a light microscope. In the image analysis process, we utilized ImagineJ software. After converting images to 8-bit format, we adjusted the threshold to isolate cell nuclei. Overlapping nuclei were separated using the “Process-Binary-Watershed” function. We automated particle analysis with the “Analyze-Analyze Particles” tool. To minimize errors, we cross-referenced results with color images, ensuring all visible cells were counted.

### Flow cytometry analysis

Cells were treated with Afzelin or transfected with NQO2 for 48 h and collected in a centrifuge tube. The cells were washed with PBS and digested with trypsin cell digestion solution. After centrifugation at 1000 g for 5 min, cells were collected, resuspended and counted to separate 5 × 10^4^ cells. The 5 × 10^4^ cells cells were centrifuged at 1000 g for 5 min and resuspended in Annexin V-FITC binding solution (Beyotime). Next, the cells were mixed with Annexin V-FITC and propidium iodide staining solution and incubated for 20 min at room temperature in the dark. After incubation, the apoptosis rate was measured using the BD C6 Plus flow cytometer (Becton, Dickinson and Company). The BD C6 Plus is a flow cytometry system that allows the measurement and analysis of various cellular parameters, including fluorescence signals emitted by labeled cells. Data acquisition and analysis was performed using BD Accuri C6 PLUS software.

### Western blot analysis

The cells were either treated with Afzelin for 48 h or transfected with NQO2. Subsequently, the total protein of A549 and H1299 cells was isolated using RIPA lysis buffer (Beyotime), and the total protein concentration was determined using the BCA kit (Beyotime). Total protein, equivalent to 30 μg, was separated with 10% SDS-PAGE and transferred to PVDF membrane. The membrane was blocked with 5% skin milk and incubated with primary antibody (Bcl-2: ab182858, Abcam; Bax: ab32503, Abcam; cleaved caspase-3: ab32042, Abcam; p- PERK: #3179, CST; p-eIF2α: #3398, CST; GRP78: 11587–1- AP, Proteintech; CHOP: #5554, CST; CRT: #12238, CST; HMGB1: ab18256, Abcam; NQO2: ab137612, Abcam; GAPDH: ab181603, Abcam) overnight at 4 °C. Subsequently, the membrane was incubated with Goat Anti-Rabbit IgG H&L (HRP) (ab205718, Abcam) for 2 h. GAPDH, a housekeeping protein, was used as an internal control. Bands were visualized using the ECL kit (Beyotime) and analyzed using Image-Pro Plus software.

### Detection of ATP, HMGB1, and CRT

After treatment with Afzelin for 48 h or transfection with NQO2, the supernatant was centrifuged for assessment of ATP, HMGB1, and CRT levels using the ATP Assay Kit (Beyotime), the ELISA Kit for HMGB1 (Wuhan USCN Business Co., Ltd.), and the Human Calreticulin ELISA Kit (Invitrogen), respectively.

### qRT-PCR analysis

Total RNA, from A549 and H1299 cells, was extracted with Trizol reagent (Beyotime) and transcribed into cDNA using the PrimeScript RT kit (Takara). qPCR was performed using BeyoFastTM SYBR Green qPCR Mix (Beyotime). GAPDH, a housekeeping gene, was used as an internal reference. Relative NQO2 mRNA expression was calculated using the 2^−ΔΔCT^ method. The primer sequences were as follows: NQO2 forward primer: 5'-GCTGGTCGGAAGATTGCTGG-3' and reverse primer: 5'-CCTGCCTGCTCAGTTCATCT-3'; GAPDH forward primer: 5'- GCTCTCTGCTCCTCCTGTTC-3' and reverse primer: 5'- GCAGGAGGCATTGCTGATGA-3'.

### Immunofluorescence

Cells were treated with Afzelin for 48 h or transfected with NQO2 and then seeded onto slides and incubated overnight. After washing with PBS, cells were fixed with 4% paraformaldehyde and permeabilized using 0.1% Triton X-100 followed by blocking of non-specific sites with 1% BSA. Cells were then incubated overnight with anti-CRT antibody (#12,238, CST) at 4 °C and Goat Anti-Rabbit secondary antibody H&L (ab150077, Abcam) for 2 h at 25 °C. Fluorescence was analyzed using a fluorescence microscope. In the image analysis process, we utilized ImagineJ software. CRT and DAPI cell counts were obtained separately, and the merged image count was derived by calculating the CRT-to-DAPI ratio multiplied by 100.

### Differential Scanning Calorimetry (DSC)

The DSC experiment was used to confirm the change in thermal transition point (Tm) after the combination of protein and small molecule i.e., Afzelin. Briefly, the thermal behavior was studied using a differential scanning calorimeter (PerkinElmer) at a heating rate of 10 °C/min. Measurements were made in the heating range of 20–250 °C in N_2_ [22, 23].

### Bioinformatic analysis

To elucidate the potential targets of Afzelin, we employed the SwissTargetPrediction tools (http://swisstargetprediction.ch/) to analyze its target genes. Subsequently, we conducted Gene Ontology (GO) analysis and pathway enrichment using the String database (https://string-db.org), utilizing the enriched genes identified from the SwissTargetPrediction analysis results. To visualize and present the results of the GO analysis and pathway enrichment, we utilized the R package ggplot2. Molecular docking between Afzelin and NQO2 was performed using Molecular Operating Environment software (MOE).

### Statistical analysis

Data is presented as mean ± SD and has been analyzed with GraphPad Prism 7.0. The difference between any two groups was analyzed with Student’s t test. Differences between multiple groups were analyzed with one-way ANOVA. A *P* value of less than 0.05 was considered statistically significant.

## Results

### Afzelin inhibits cell proliferation and promotes apoptosis of lung cancer cells

CCK-8 assay showed that Afzelin treatment significantly reduced cell viability of A549 and H1299 cells at concentrations ranging from 10 to 40 μM after 48 h (Fig. 1B). In A549 cells, the IC50 values decreased from 68.41 μM at 24 h to 35.47 μM at 48 h and further to 14.07 μM at 72 h. Similarly, in H1299 cells, the IC50 values decreased progressively from 133.44 μM at 24 h to 46.92 μM at 48 h and ultimately to 16.30 μM at 72 h. The CCK-8 experiment was employed to investigate the impact of Afzelin on normal lung epithelial cells. Notably, the results demonstrated that Afzelin did not significantly inhibit the cell viability of 16HBE cells (Figure S1A). Simultaneously, Afzelin demonstrated a lack of toxicity to 16HBE cells at both 24 h (IC50: 211.0042 μM) and 48 h (IC50: 152.2603 μM). Only at 72 h (IC50: 131.3185 μM) did a mild cytotoxic effect become apparent. Colony formation analysis showed that Afzelin treatment drastically reduced the number of colonies (Fig. 1C). In parallel experiments, treatment with Afzelin at concentrations of 10–40 μM significantly accelerated apoptosis (Fig. 1D). In addition, Afzelin treatment significantly decreased Bcl-2 expression while increase in Bax and cleaved caspase-3 levels were observed in A549 and H1299 cells (Fig. 1E).

### Afzelin induces ER stress and ICD in lung cancer cells

**Fig. 2:**
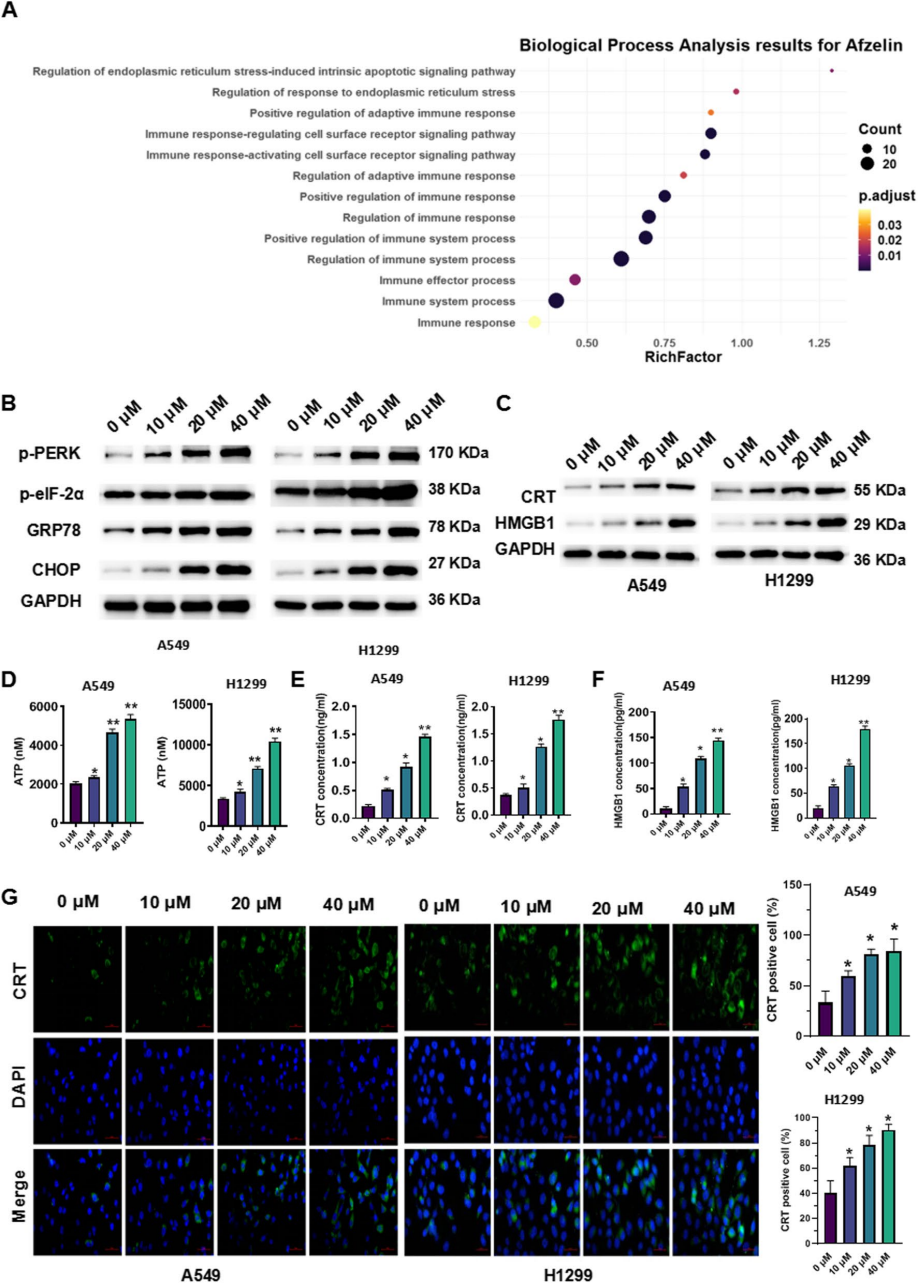
Afzelin induces ER stress and ICD in lung cancer cells. **A** The biological process analysis for afzelin using String, **B** expression of ER stress-related proteins, and **C** ICD markers in A549 and H1299 cells. The levels of (**D**) ATP, (**E**) CTR, and (**F**) HMGB1. **G** CTR expression was assessed by immunofluorescence. *P < 0.05, **P < 0.01

SwissTargetPrediction (http://swisstargetprediction.ch/) was employed to predict the target gene of Afzelin. Subsequently, the web tool String (https://string-db.org) was used for GO analysis and pathway enrichment based on these target genes (Fig. 2A). Afzelin treatment significantly increased the levels of p-PERK, p-eIF-2α, GRP78, and CHOP in A549 and H1299 cells (Fig. 2B). In addition, Afzelin treatment significantly increased the protein levels of the ICD markers, ATP, CRT and HMGB1 (Fig. 2C). Afzelin treatment markedly increased the concentrations of ATP, CRT, and HMGB1 in the supernatant, indicating that Afzelin induces the release of ATP, CRT, and HMGB1 (Fig. 2D-F). Immunofluorescence experiments suggested that Afzelin promotes the transfer of CRT to the cell membrane (Fig. 2G).

### NQO2 is identified as a potential target of Afzelin in lung cancer cells

**Fig. 3:**
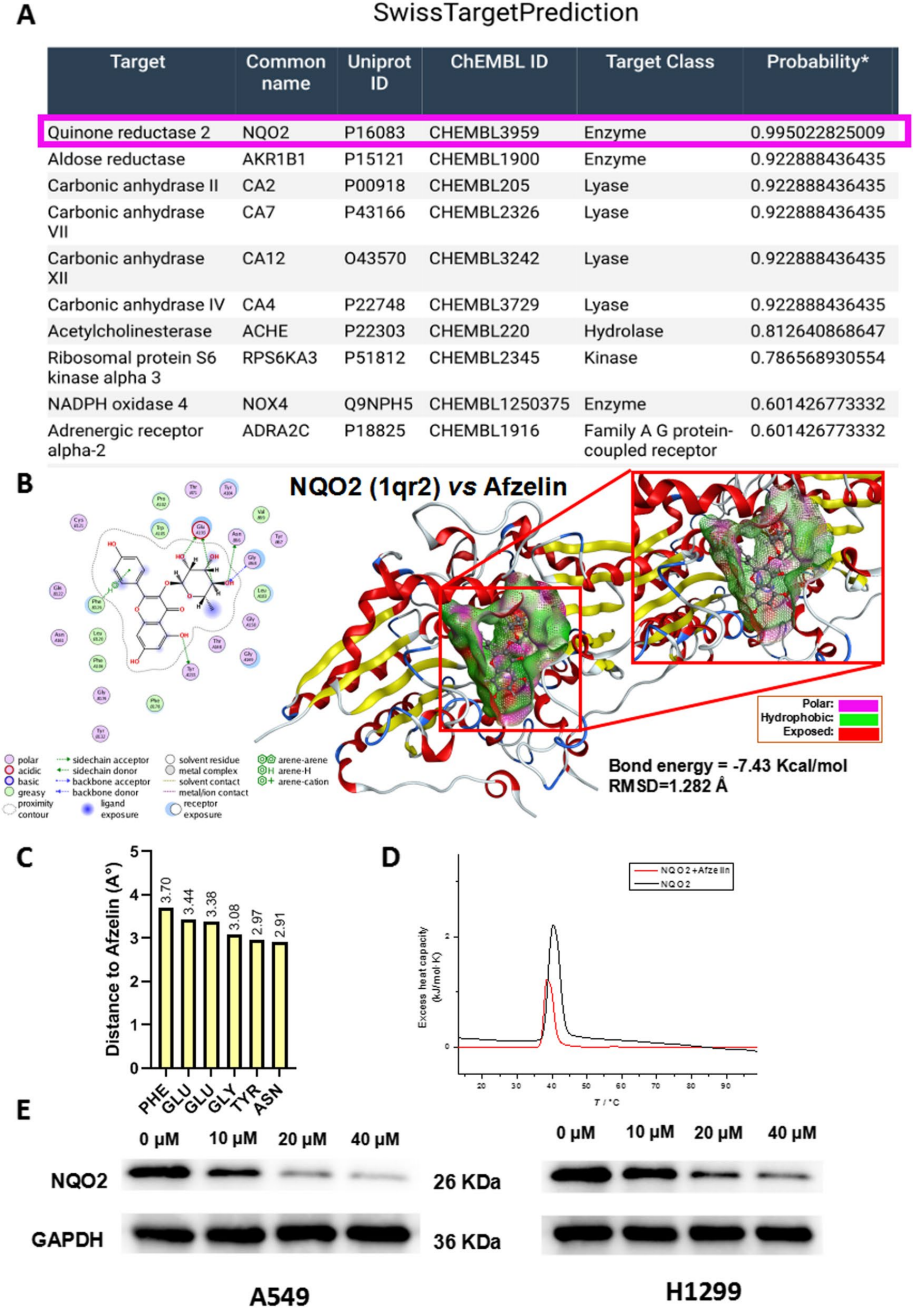
NQO2 is identified as a potential target of Afzelin in lung cancer cells. **A** The target gene of Afzelin was predicted using SwissTargetPrediction. **B** Molecular docking between Afzelin and NQO2 was performed using MOE software. **C** The distance between Afzelin and the predicted interacting amino acids is shown. **D** The T_m_ for NQO2 was determined using DSC. **E** The NQO2 protein expression in A549 and H1299 cells is shown

NQO2, ranked first in SwissTargetPrediction as a target of Afzelin, and, was selected for further analysis and molecular docking between Afzelin and NQO2 was performed using MOE (Fig. 3A-B). The distance between Afzelin and predicted interacting amino acids was analyzed (Fig. 3C) and the change in the mean melting temperature (T_m_) of NQO2 was investigated using DSC. The T_m_ was significantly lower for NQO2 bound to Afzelin (Fig. 3D). In addition, NQO2 protein expression after Afzelin treatment showed a significant decrease (Fig. 3E).

### Afzelin regulates cell proliferation and apoptosis of lung cancer cells via NQO2

**Fig. 4:**
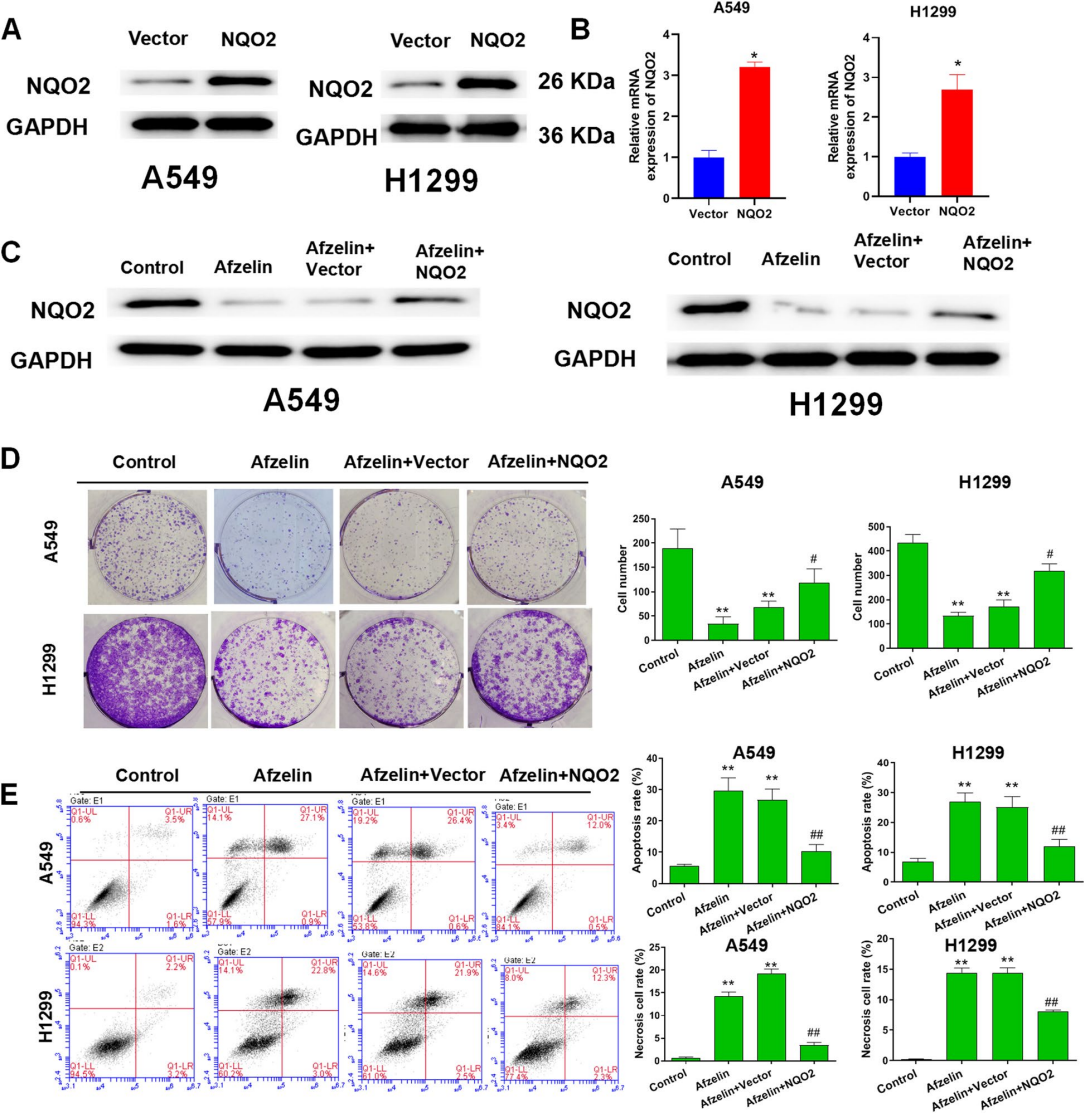
Afzelin inhibits proliferation and promotes apoptosis of lung cancer cells via NQO2. **A** NQO2 protein and **B** its mRNA expression are shown. The NQO2 protein expression (**C**), proliferation (**D**), and apoptosis (**E**) of A549 and H1299 cells is visible. *P < 0.05, **P < 0.01, ***P < 0.001 vs. control group. #*P* < 0.05, ##*P* < 0.01 vs. Afzelin plus Vector group

NQO2 overexpression plasmid was transfected into Afzelin-treated A549 and H1299 cells. Western blot and qRT-PCR showed that NQO2 overexpression significantly increased NQO2 levels (Fig. 4A and B). Evidently, Afzelin significantly decreased the NQO2 protein level in comparison to the control group. However, the decreased level of NQO2 protein was significantly restored by NQO2 overexpression (Fig. 4C). The colony formation analysis showed that Afzelin significantly inhibited cell proliferation. Overexpression of NQO2 markedly promoted proliferation, previously inhibited by Afzelin (Fig. 4D). Flow cytometry showed that Afzelin accelerated apoptosis. NQO2 overexpression reduced apoptosis in Afzelin enhanced apoptotic cells (Fig. 4E). These data suggest that overexpression of NQO2 can reverse the effect of Afzelin on proliferation and apoptosis of A549 and H1299 cells.

### Afzelin induces ER stress and ICD via NQO2

**Fig. 5:**
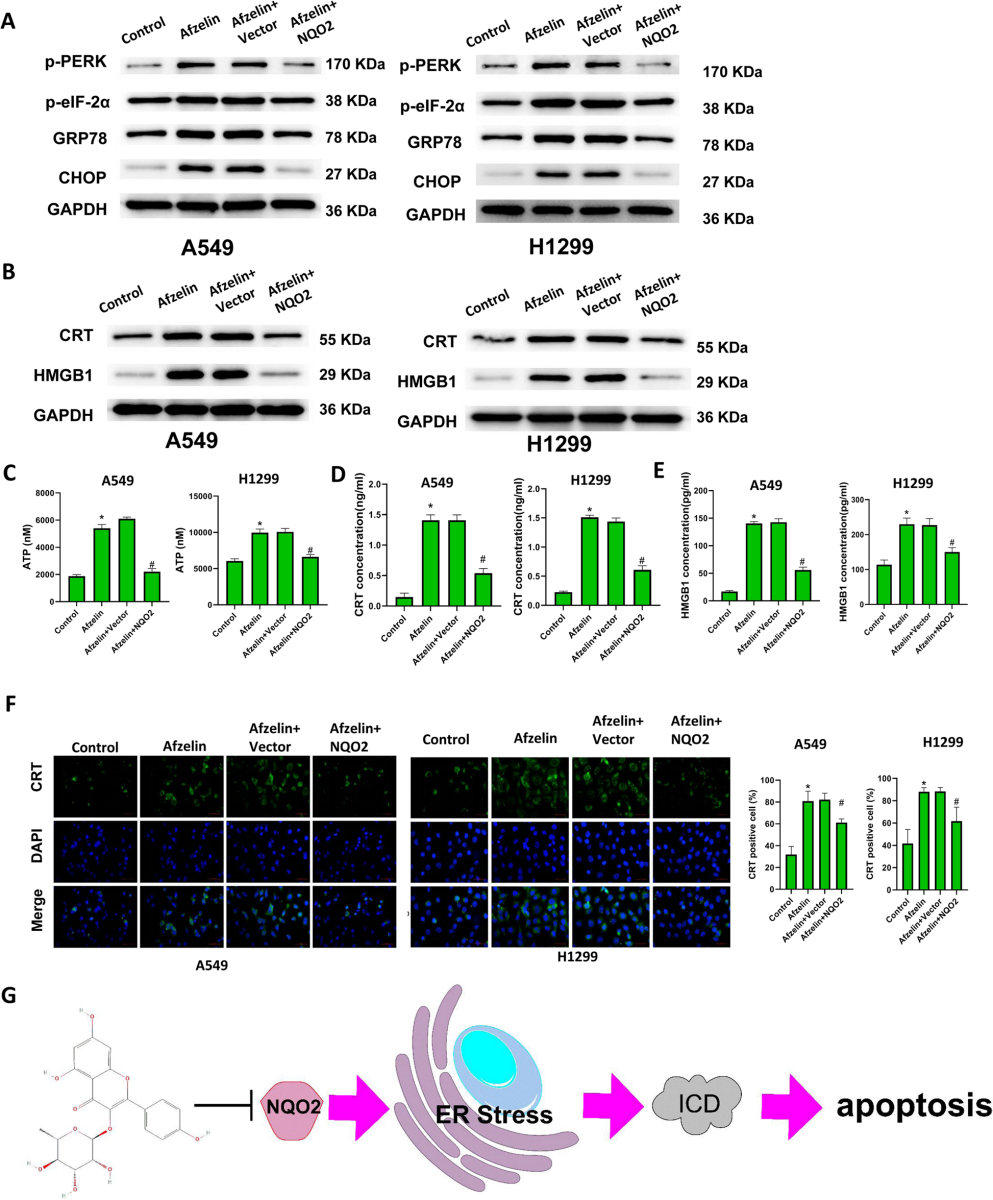
Afzelin induces ER stress and ICD via NQO2. **A** ER stress-related proteins, **B** CRT and HMGB1, **C** ATP, **D** CTR, and **E** HMGB1 expression in A549 and H1299 cells is shown. **F** The expression of CTR in A549 and H1299 cells was examined by immunofluorescence. **G** A schematic model of NQO2 function in lung cancer. *P < 0.05 vs control group. #*P* < 0.05 vs. Afzelin plus Vector group

As shown in Fig. 5A, Afzelin treatment significantly increased the levels of ER stress-related proteins in A549 and H1299 cells. NQO2 overexpression decreased Afzelin induced ER stress protein levels. Similarly, levels of CRT and the ICD markers, ATP and HMGB1, were significantly increased after Afzelin treatment and reversed by NQO2 overexpression (Fig. 5B-F). A schematic model of NQO2 function in lung cancer was shown in Fig. 5G.

## Discussion

Lung cancer has the highest mortality rate, posing a major threat to people’s health and a huge economic burden on society [1]. Although new treatments have been developed in the past decades, the survival rate of lung cancer patients remains low due to metastases, recurrences or complications [24, 25]. The current study reports that Afzelin inhibits cancer cell proliferation in vitro and induces apoptosis. Further investigation of the mechanism of Afzelin-mediated apoptosis showed that Afzelin specifically inhibits NQO2 expression, thereby activating ER stress and ICD, and ultimately triggering apoptosis.

ICD is a newly reported form of regulated cell death that triggers an adaptive immune response to cancer cells [26]. CRT exposure, HMGB1 and ATP release are known as ICD markers [27, 28]. HMGB1 is passively released from necrotic or damaged cells and transmits a signal for cell death [29]. The extracellularly secreted ATP binds to the receptor on the target cell and attracts macrophages [30]. CRT is a soluble protein in the ER cavity that reaches the cell surface at the pre-death stage and activates phagocytic cells to effectively phagocytose dead cells [31]. In this study, the levels of ATP, CRT, and HMGB1 were significantly increased after Afzelin treatment. Immunofluorescence studies displayed that CRT translocated from intracellular space to membrane after Afzelin treatment. The above results suggest that Afzelin treatment induced ICD in lung cancer cells. Induction of ICD in cancer cells can activate the immune system, thereby inhibiting breast cancer progression [32]. This induction has been reported to effectively inhibit metastasis and tumor growth in gastric cancer [33]. In this study, Afzelin promoted apoptosis of lung cancer cells by inducing ICD, which is also consistent with previous reports.

Furthermore, ICD is divided into two modes according to its induction mechanism: I ICD i.e., an indirect signal and II ICD i.e., direct signal inducing ER stress [34]. Phosphorylation of eIF2α by activated PERK (p-PERK) is the hallmark of ER stress [35–37]. Under ER stress, p-PERK phosphorylates EIF2α to p-eIF2α, leading to high expression of the pro-apoptotic transcription factor, CHOP [38]. The chaperone protein, GRP78, is the major regulator of ER homeostasis [39]. In this study, bioinformatic analysis revealed that Afzelin is involved in ER stress. Afzelin promoted the phosphorylation of PERK and EIF2 α and upregulated the protein levels of GRP78 and CHOP. Studies have shown that triggering ER stress can induce lung cancer cell death [40]. Chen P et al. have showed that the induction of ER stress can trigger the death of lung cancer cells induced by Lathyrol [41]. The current results are consistent with the above study and suggest that Afzelin promotes apoptosis of lung cancer cells via activation of ER stress.

In addition, bioinformatic analysis revealed that NQO2 was the target of Afzelin. NQO2, a flavin adenine mononucleotide-dependent quinone oxidoreductase, is ubiquitously found in various tissues such as liver, kidney, brain, heart, and lung [42]. Earlier studies have shown that NQO2 may accelerate the inactivation of cancer-causing orthoquinones and thus, contribute to breast cancer progression [43]. In addition, NQO2 knockout mouse significantly inhibits prostate cancer cell growth [44]. More importantly, elimination of NQO2 enhances apoptosis of lung cancer cells [45]. In this study, Afzelin significantly inhibited the expression of NQO2 in lung cancer cells. Meanwhile, our results also showed that overexpression of NQO2 promoted the proliferation of A549 and H1299 cells, while inhibiting apoptosis, ER stress, and ICD in A549 and H1299 cells (Figure S1B-G). This is also consistent with previous research [45]. Further study found that overexpression of NQO2 apparently attenuated the role of Afzelin in apoptosis of lung cancer cells. In addition, the overexpression of NQO2 counteracts the ER stress induced by Afzelin, resulting in reduced levels of p-PERK and p-eIF2α. This, in turn, affects the protein levels of GRP78 and CHOP, as well as the induction of ICD markers including CRT, HMGB1, and ATP caused by Afzelin. These findings provide compelling evidence that Afzelin activates ER stress and induces ICD through the selective inhibition of NQO2.

Overall, our study highlights the involvement of NQO2 in the effects of Afzelin on lung cancer cells. By targeting NQO2, Afzelin promotes ER stress activation, induces ICD, and inhibits lung cancer proliferation. These findings provide valuable insights into the potential therapeutic implications of Afzelin in lung cancer treatment. Further investigations are warranted to explore the underlying mechanisms and clinical applications of Afzelin-induced inhibition of NQO2 in lung cancer therapy.

### Supplementary Information

**Additional file 1: Figure S1.** Impact of Afzelin on 16HBE cells and NQO2 on Proliferation, Apoptosis, ER Stress, and ICD in A549 and H1299 cells. (A) The CCK-8 assay was employed to assess cell viability in normal lung epithelial cells. (B) Colony assay was performed to evaluate the formation of colonies. (C) The detection of apoptosis in A549 and H1299 cells. (D) ER stress-related proteins were examined by western blot. (E-G) ELISA kits were used to measure the levels of ATP (E), CTR (F), and HMGB1 (G) in A549 and H1299 cells. *P < 0.05 vs vector group.

**Additional file 2.**

## Data Availability

The data used to support the findings of this study are available from the corresponding author upon request.
