# Supplementary figures and images for: Afzelin induces immunogenic cell death against lung cancer by targeting NQO2
Source: BMC Complement Med Ther. 2023 Oct 27;23:381. doi: 10.1186/s12906-023-04221-3 (PMC10605937; doi:10.1186/s12906-023-04221-3)

**A**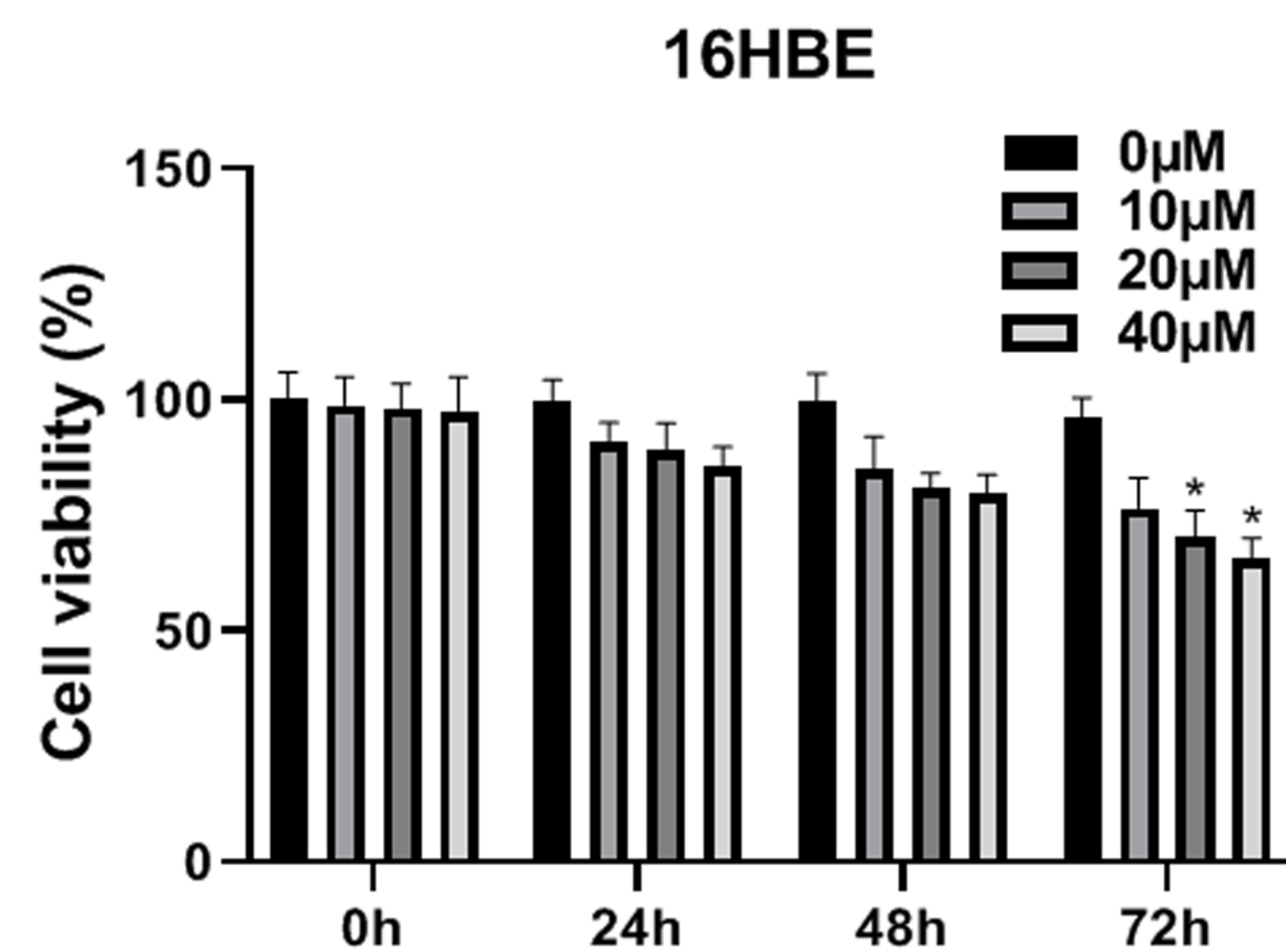**B**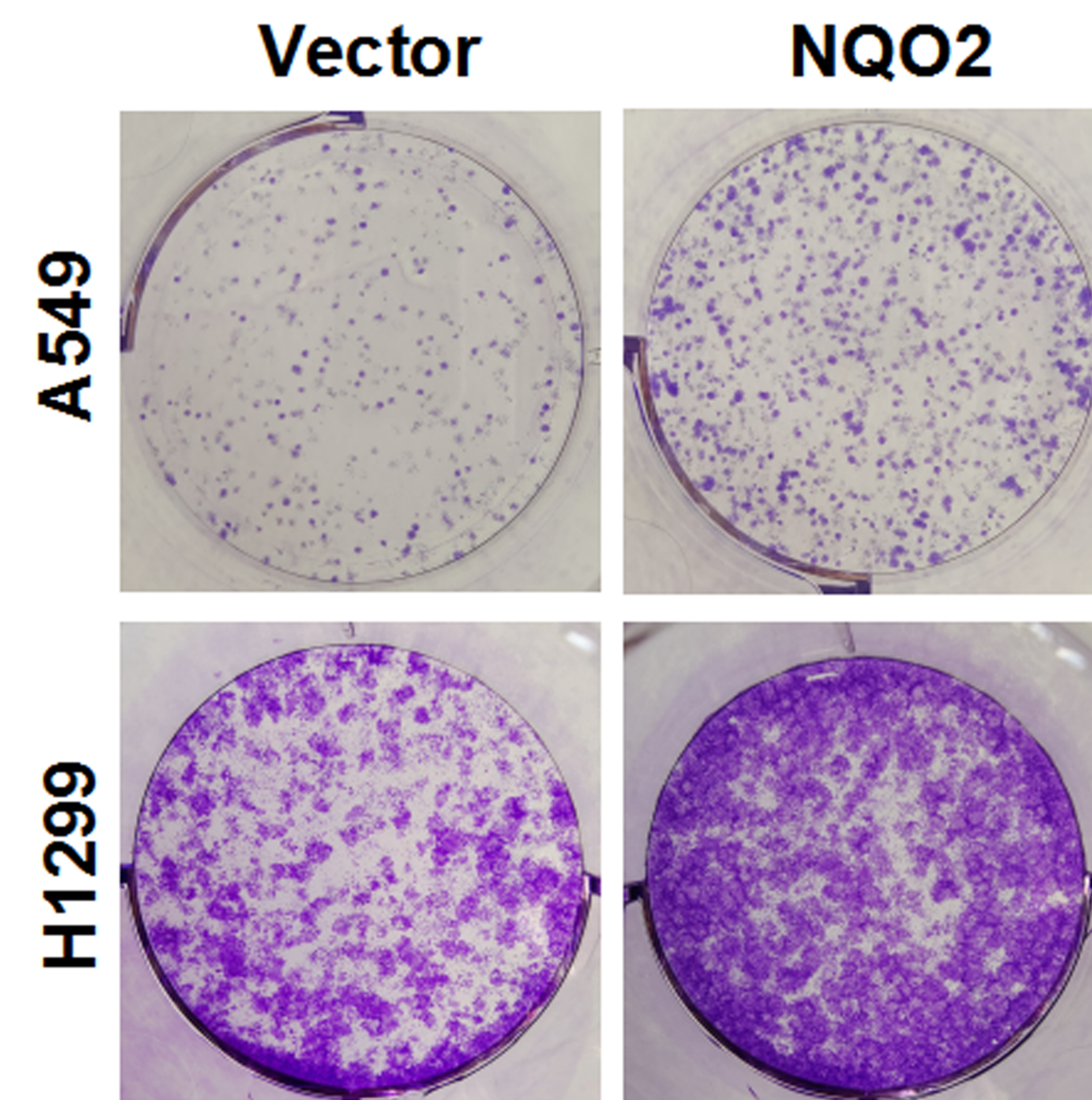**C**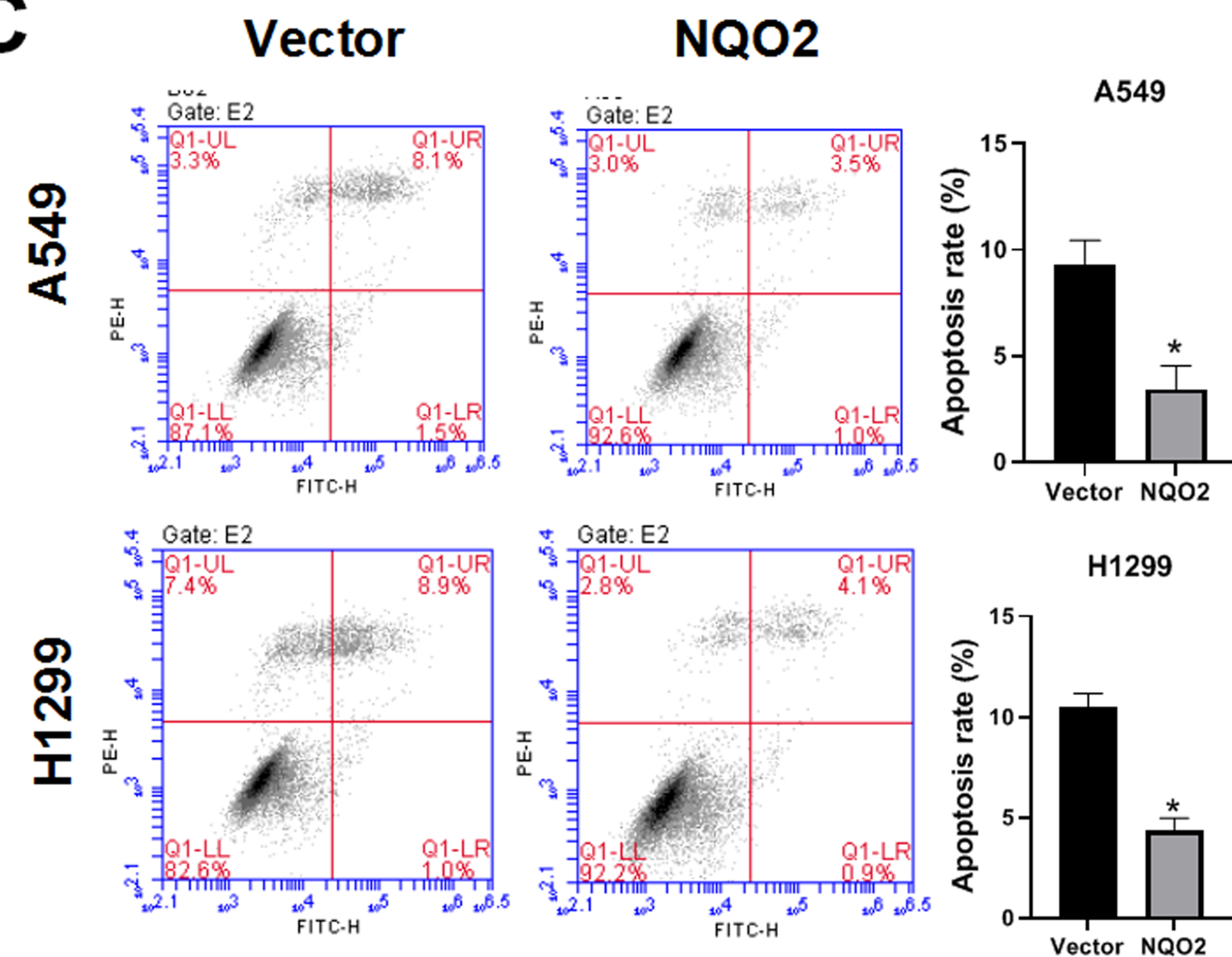**D**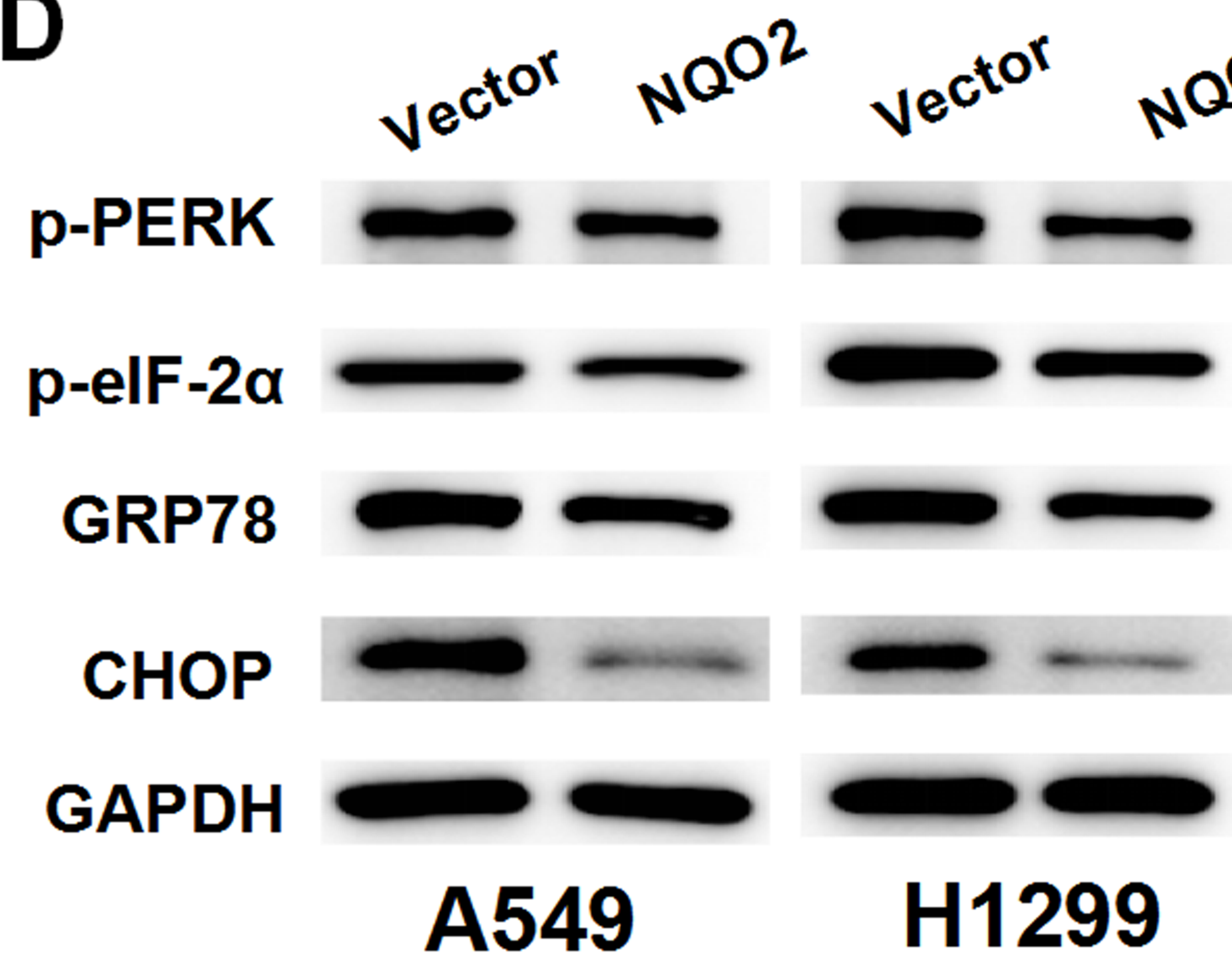**F**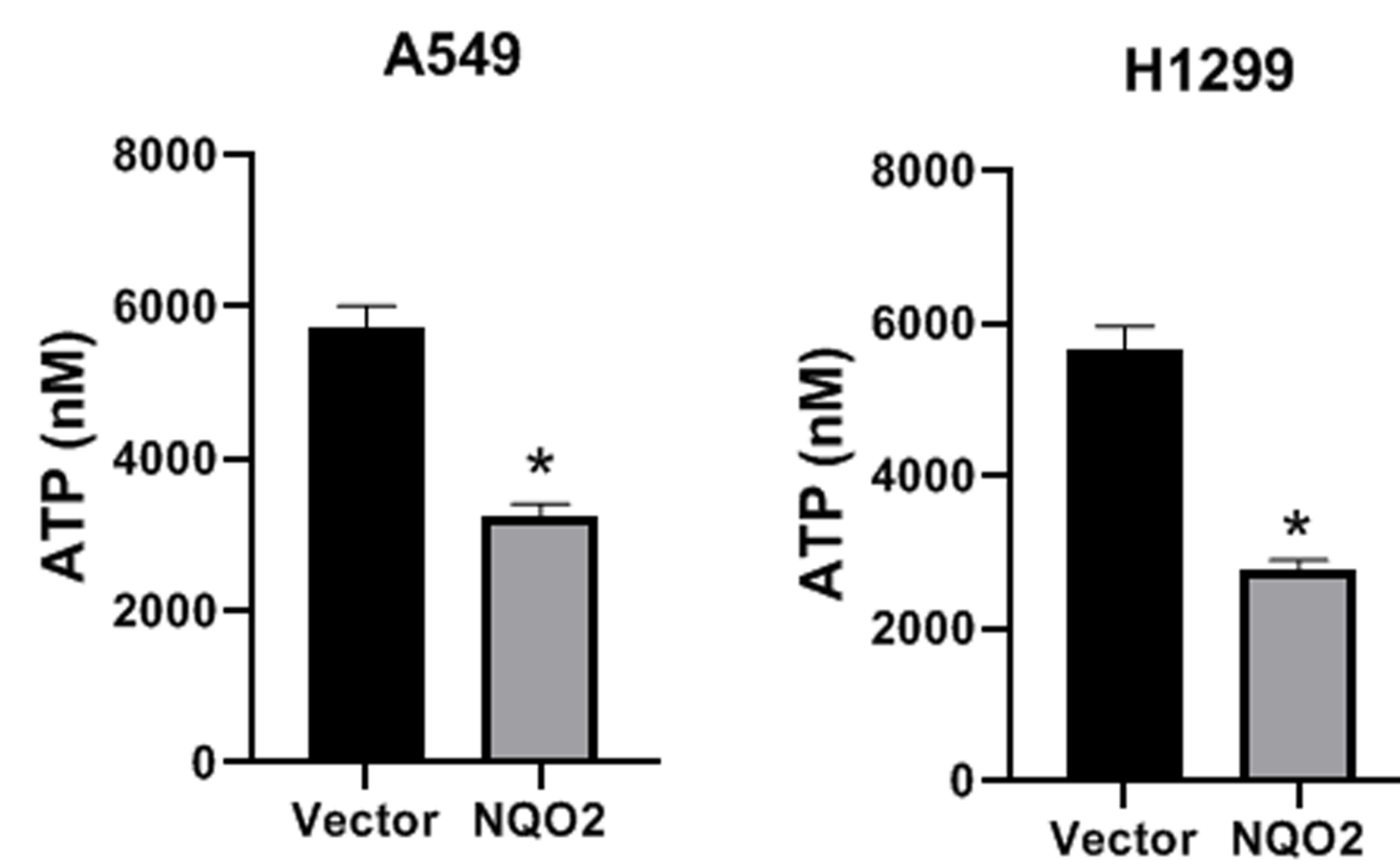**G**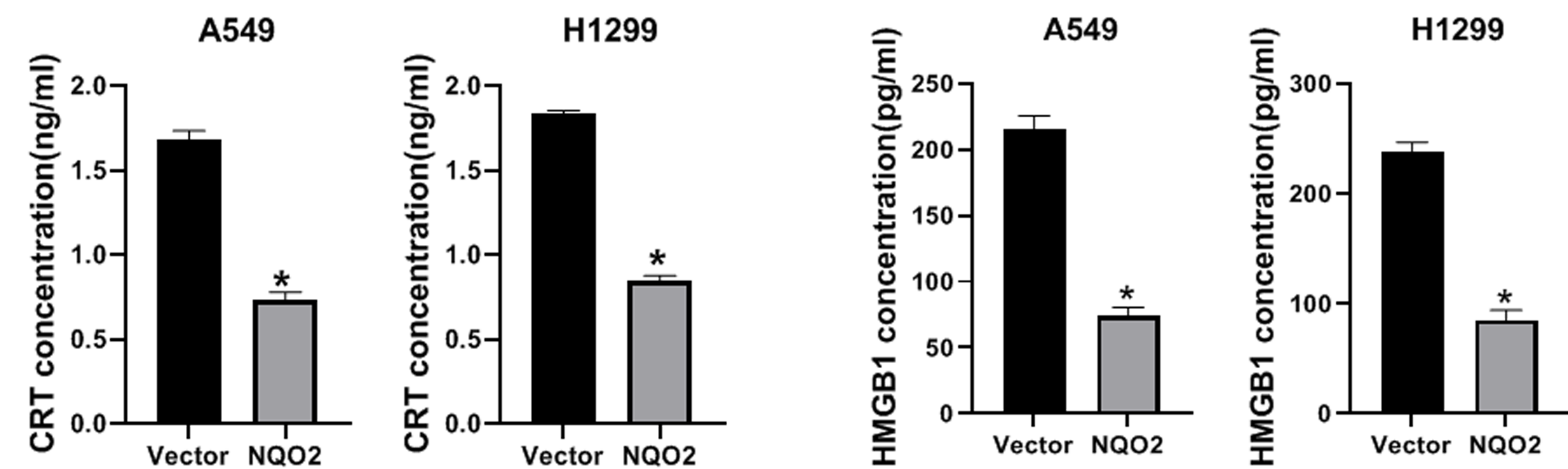

Supplement: Supplementary file 1 — Additional file 1: Figure S1. Impact of Afzelin on 16HBE cells and NQO2 on Proliferation, Apoptosis, ER Stress, and ICD in A549 and H1299 cells. (A) The CCK-8 assay was employed to assess cell viability in normal lung epithelial cells. (B) Colony assay was performed to evaluate the formation of colonies. (C) The detection of apoptosis in A549 and H1299 cells. (D) ER stress-related proteins were examined by western blot. (E-G) ELISA kits were used to measure the levels of ATP (E), CTR (F), and HMGB1 (G) in A549 and H1299 cells. *P < 0.05 vs vector group. [file 12906_2023_4221_MOESM1_ESM.pdf]
