# Supplementary material for: Afzelin induces immunogenic cell death against lung cancer by targeting NQO2
Source: BMC Complement Med Ther. 2023 Oct 27;23:381. doi: 10.1186/s12906-023-04221-3 (PMC10605937; doi:10.1186/s12906-023-04221-3)

**Figure 1E. Apoptosis-related proteins** **were examined by western blot.**

1E-A549-BAX


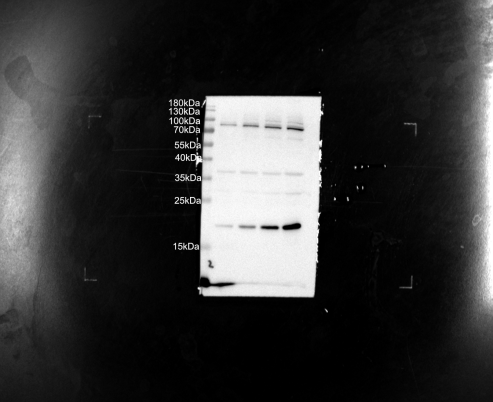


1E-A549-Bcl-2


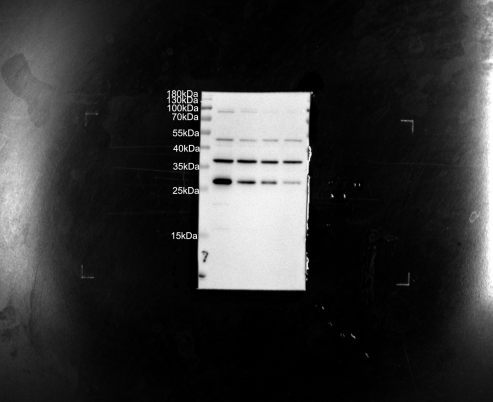


1E-A549-c-cas-3


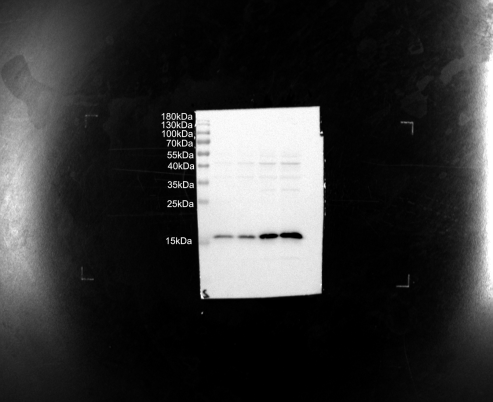


1E-A549-GAPDH


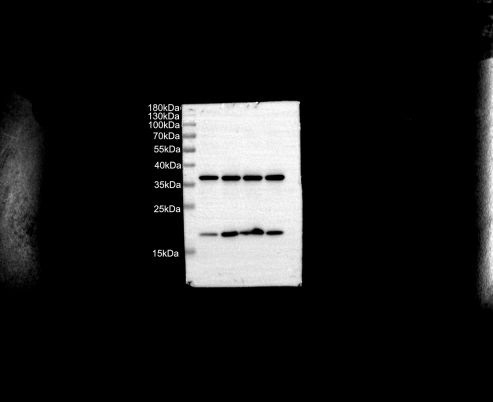


1E-H1299-BAX


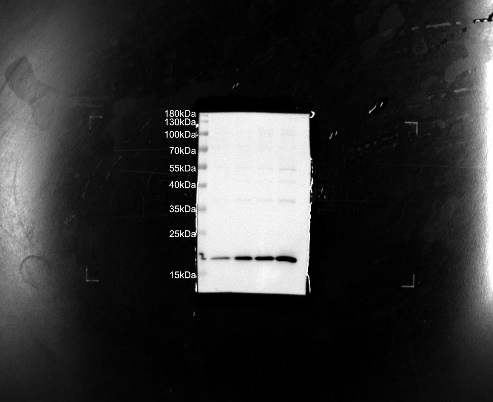


1E-H1299-Bcl-2


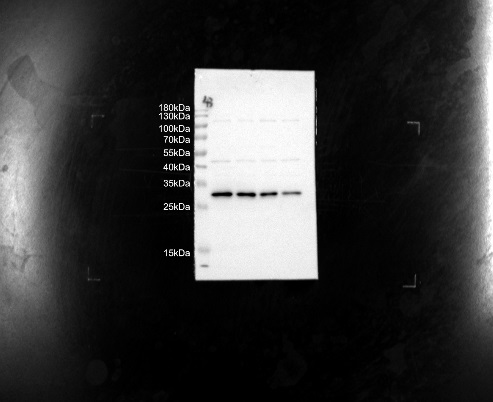


1E-H1299-c-cas-3


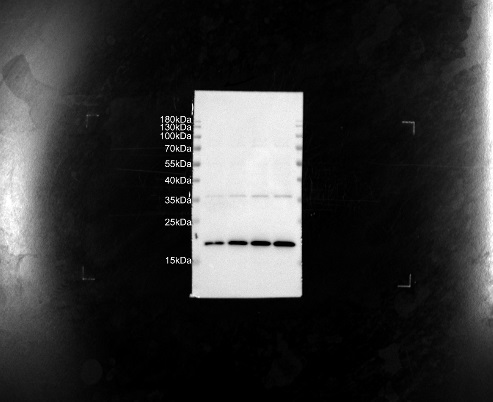


1E-H1299-GAPDH


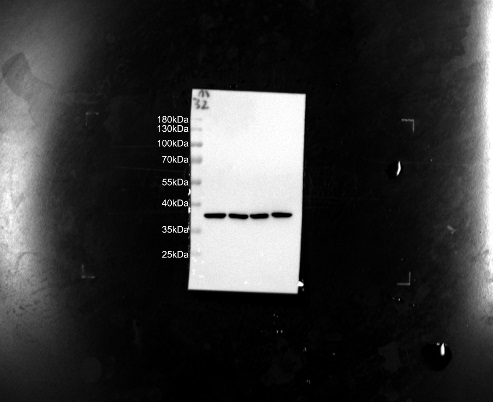


**Figure 2B. ER stress-related proteins were examined by western blot.**

2B-A549-CHOP


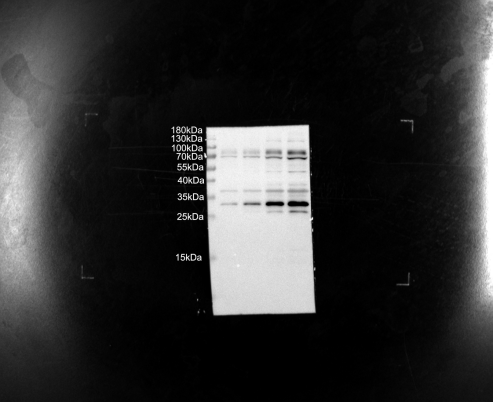


2B-A549-GAPDH


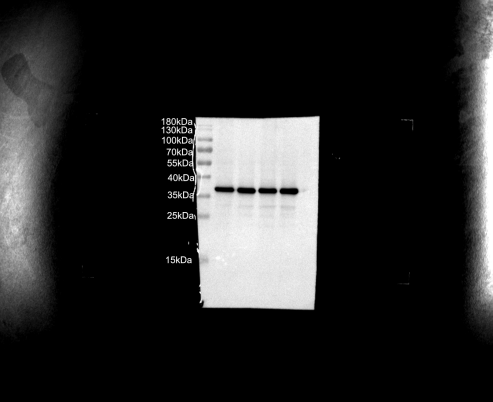


2B-A549-GRP78


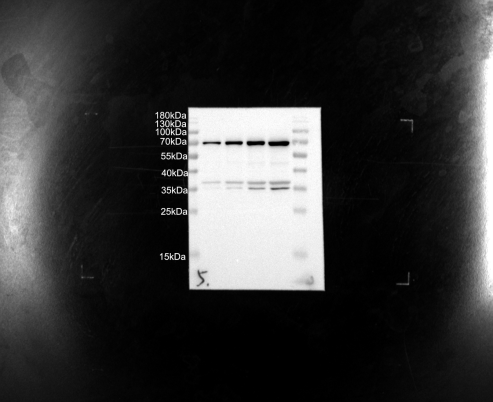


2B-A549-p-EIF2a


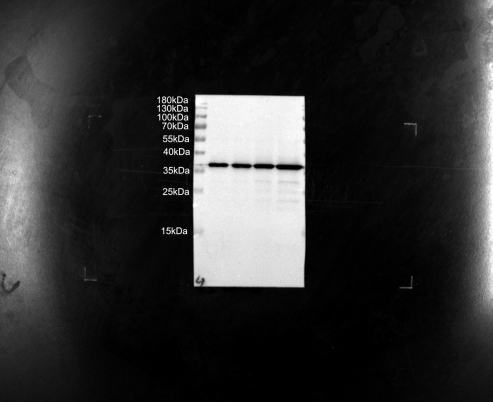


2B-A549-p-PERK


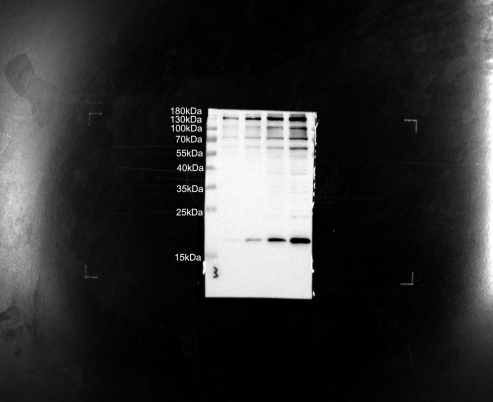


2B-H1299-CHOP


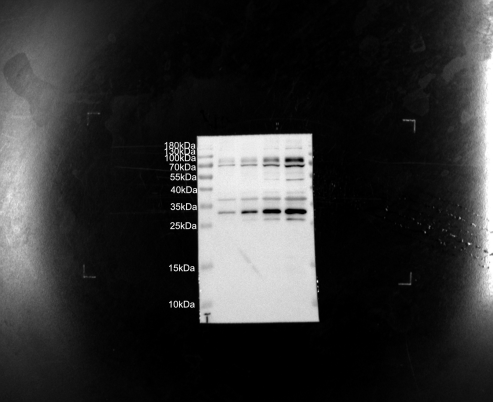


2B-H1299-GAPDH


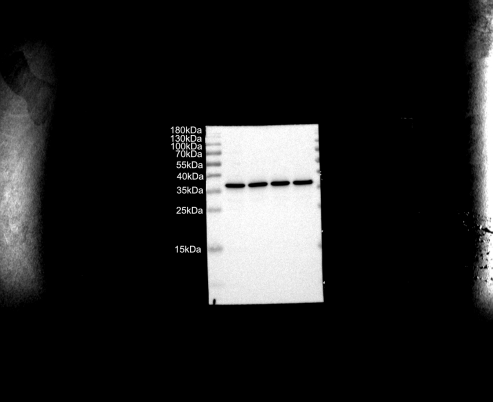


2B-H1299-GRP78


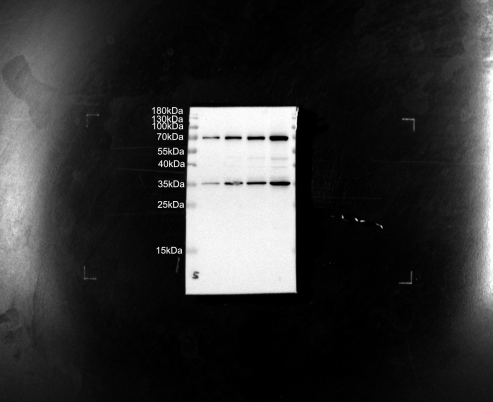


2B-H1299-p-EIF2a


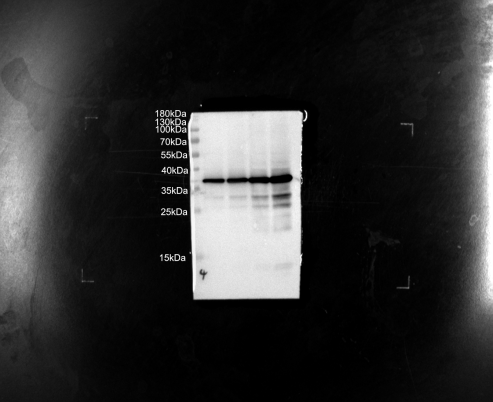


2B-H1299-p-PERK


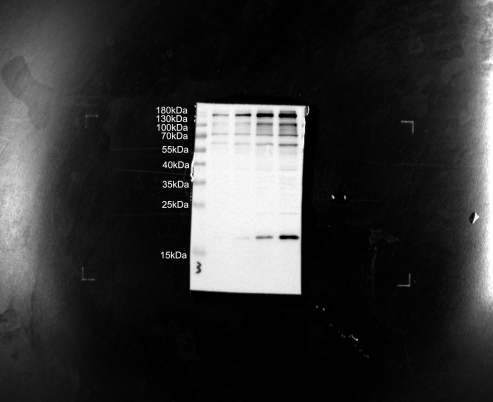


**Figure 2C. ICD-related proteins were examined by western blot.**

2C-A549-CRT


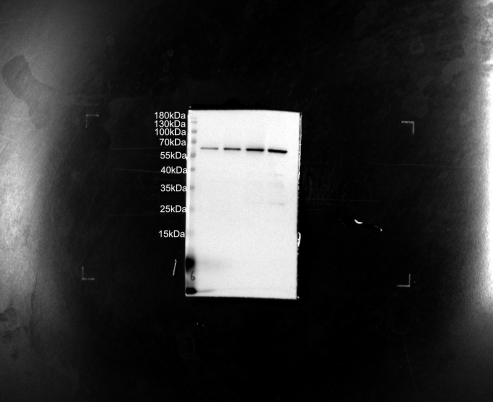


2C-A549-GAPDH


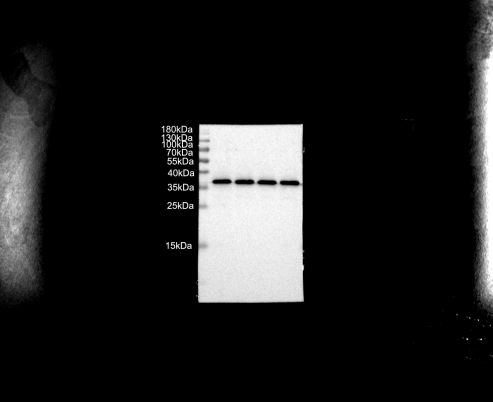


2C-A549-HMGB1


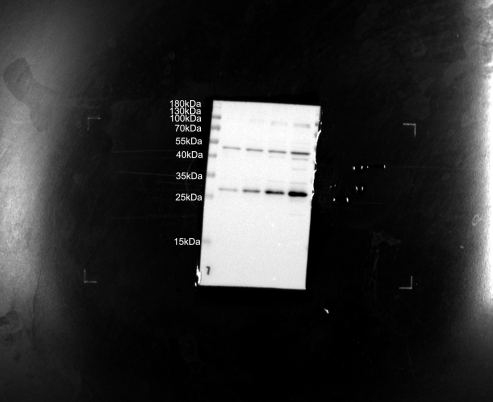


2C-H1299-CRT


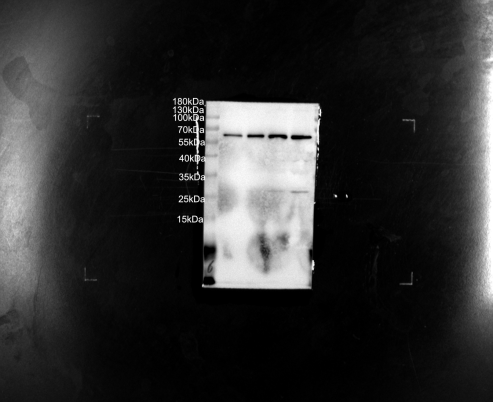


2C-H1299-GAPDH


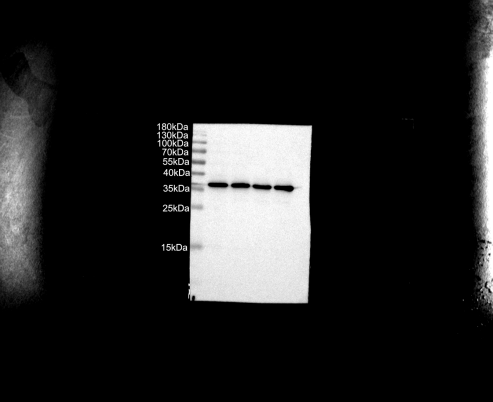


2C-H1299-HMGB1


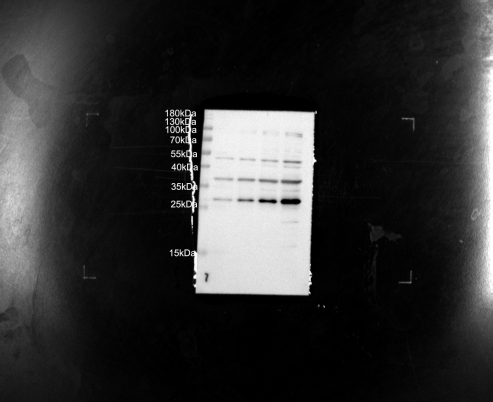


**Figure 3E. The NQO2 protein expression in A549 and H1299 cells were examined by western blot.**

3E-A549-GAPDH


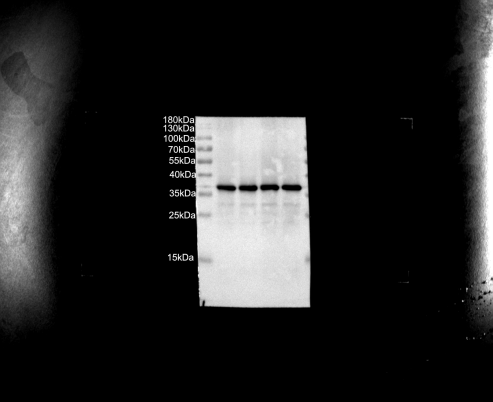


3E-A549-NQO2


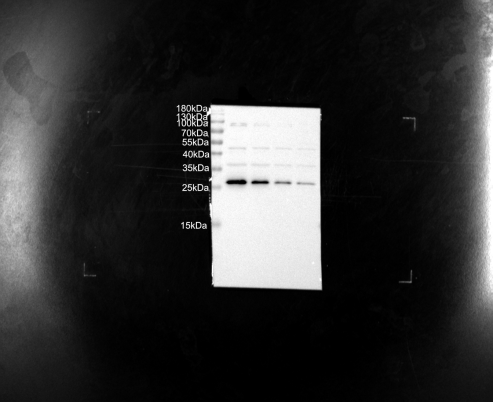


3E-H1299-GAPDH


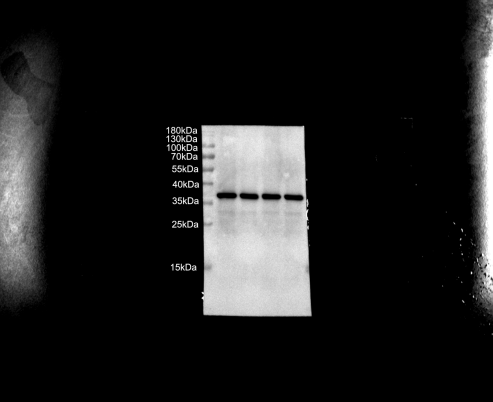


3E-H1299-NQO2


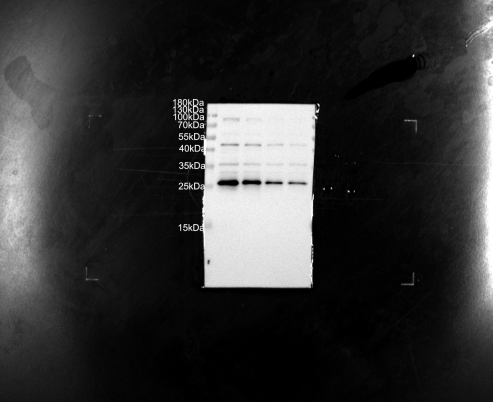


**Figure 4A. The protein expression of NQO2 in A549 and H1299 cells after overexpression was examined by western blot.**

4A-A549-GAPDH


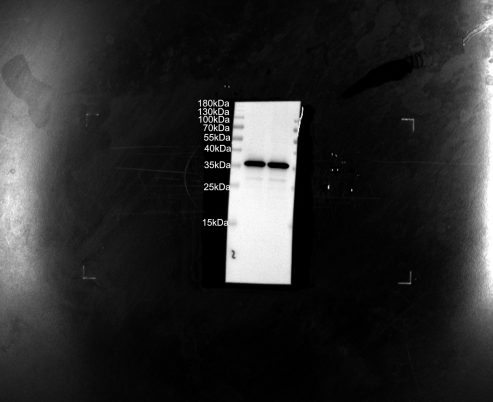


4A-A549-NQO2


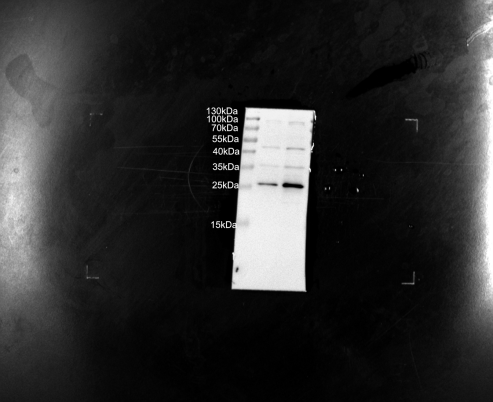


4A-H1299-GAPDH


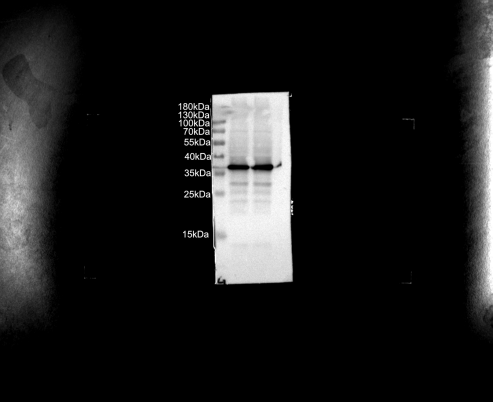


4A-H1299-NQO2


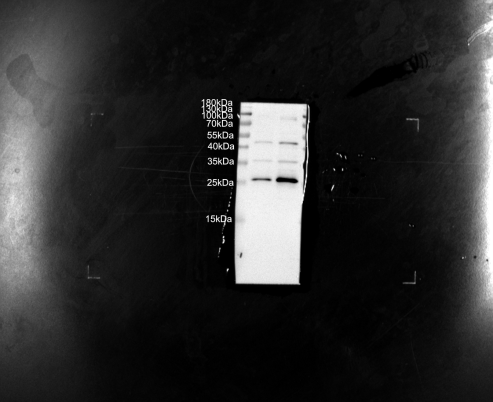


**Figure 4C. The protein expression of NQO2 in A549 and H1299 cells was examined by western blot.**

4C-A549-GAPDH


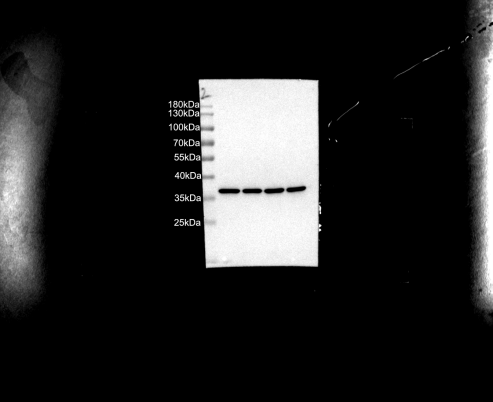


4C-A549-NQO2


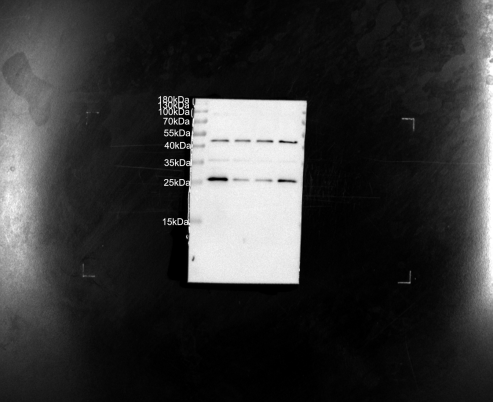


4C-H1299-GAPDH


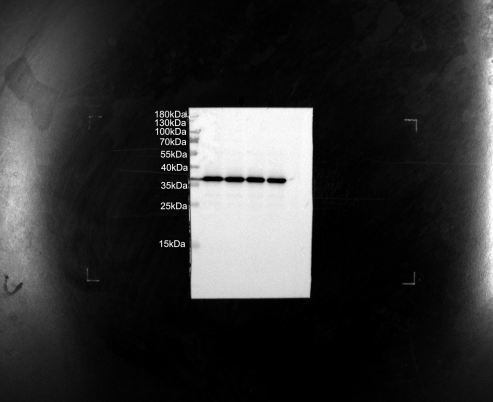


4C-H1299-NQO2


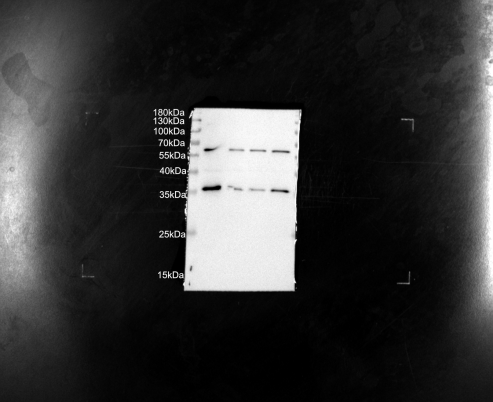


**Figure 5A. ER stress-related proteins were examined by western blot.**

5A-A549-CHOP


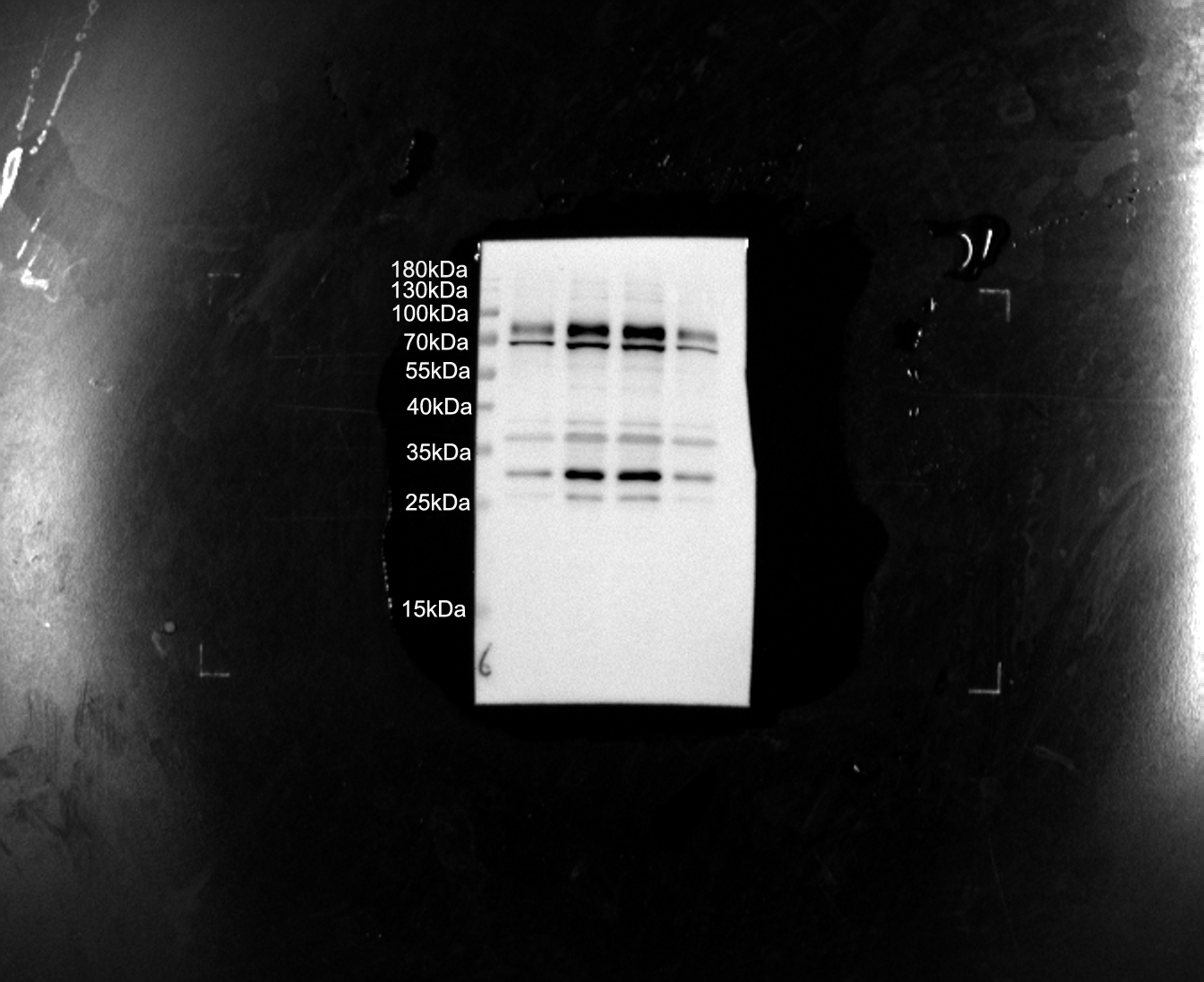


5A-A549-GAPDH


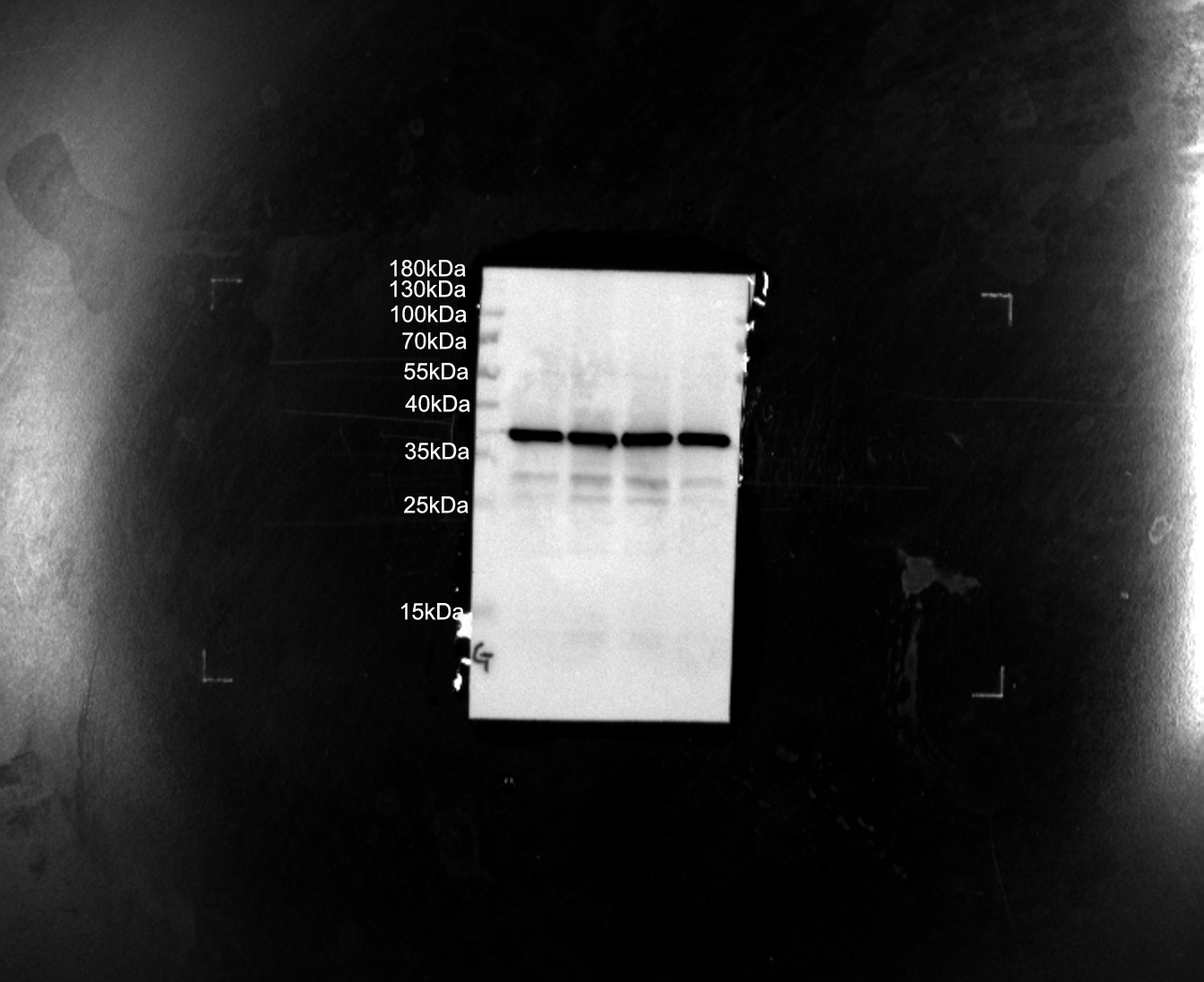


5A-A549-GRP78


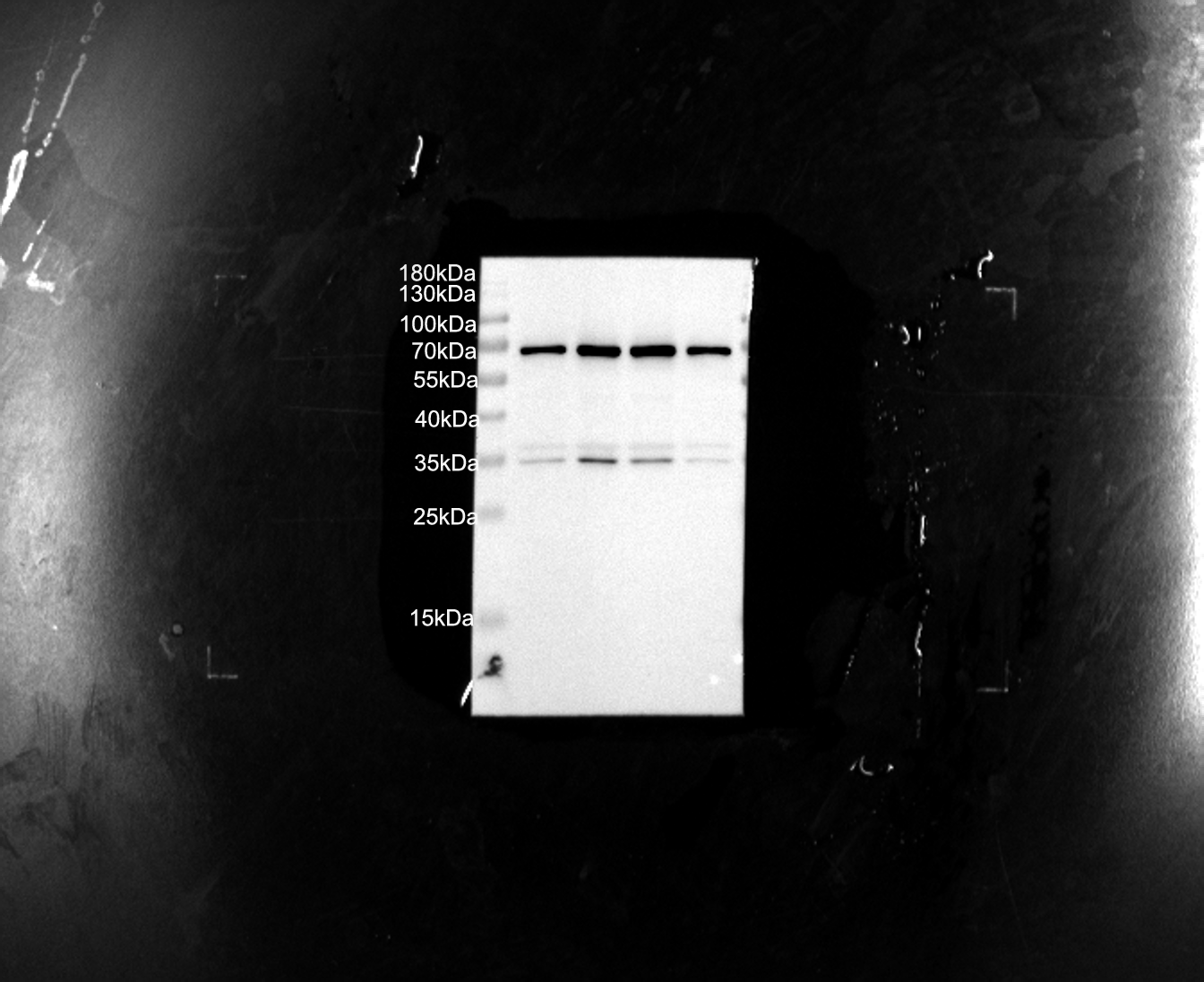


5A-A549-p-EIF2a


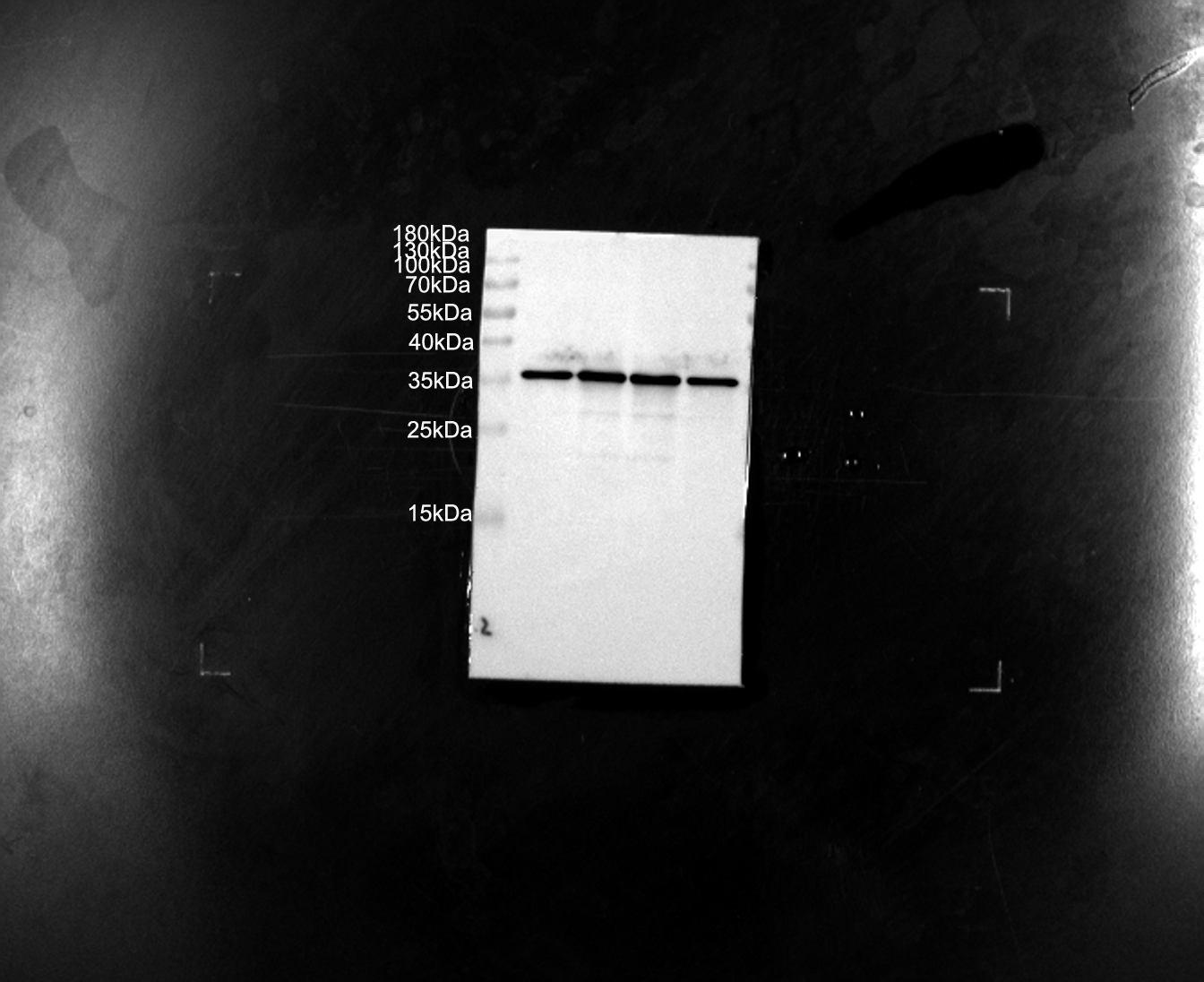


5A-A549-p-PERK


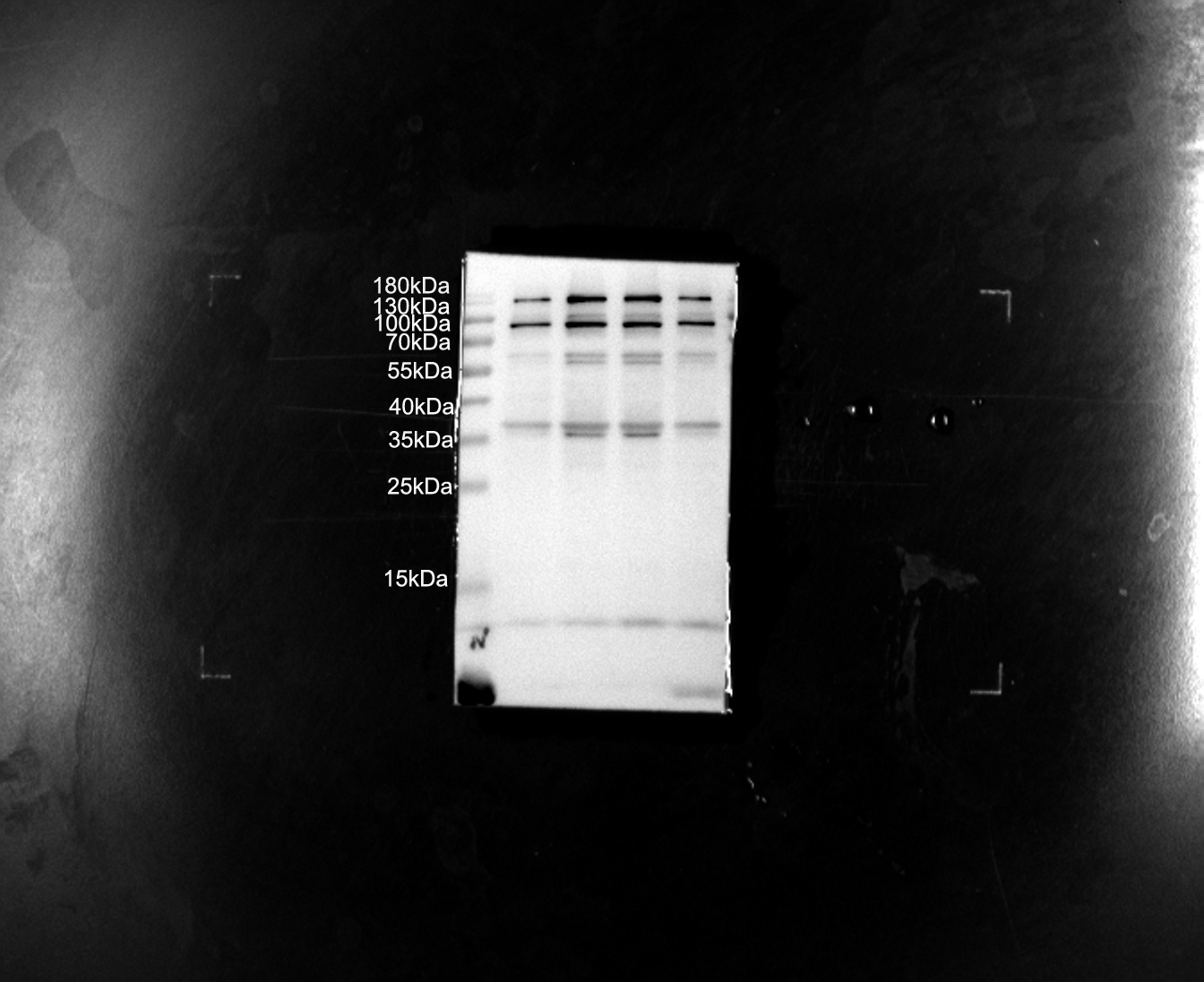


5A-H1299-CHOP


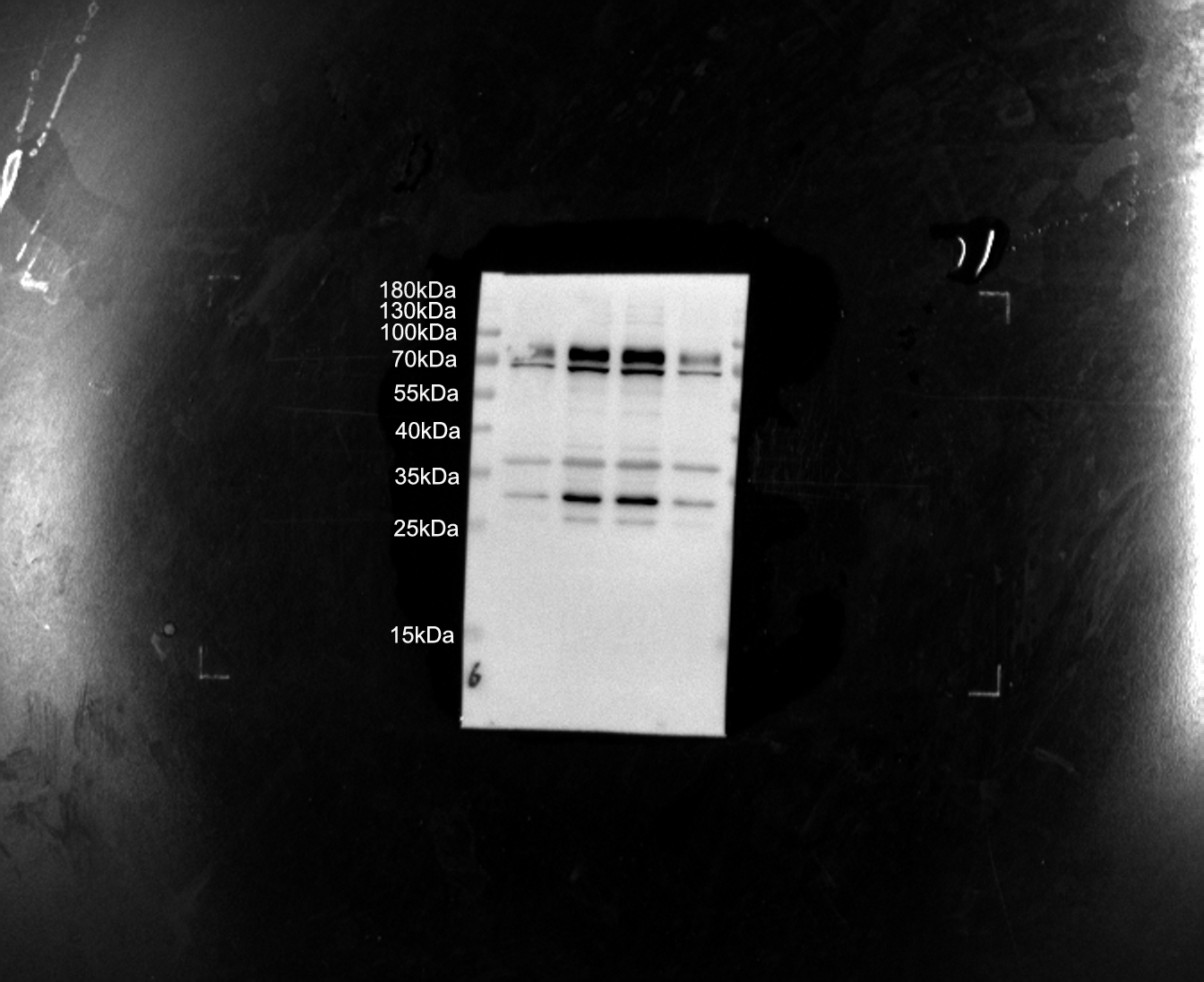


5A-H1299-GAPDH


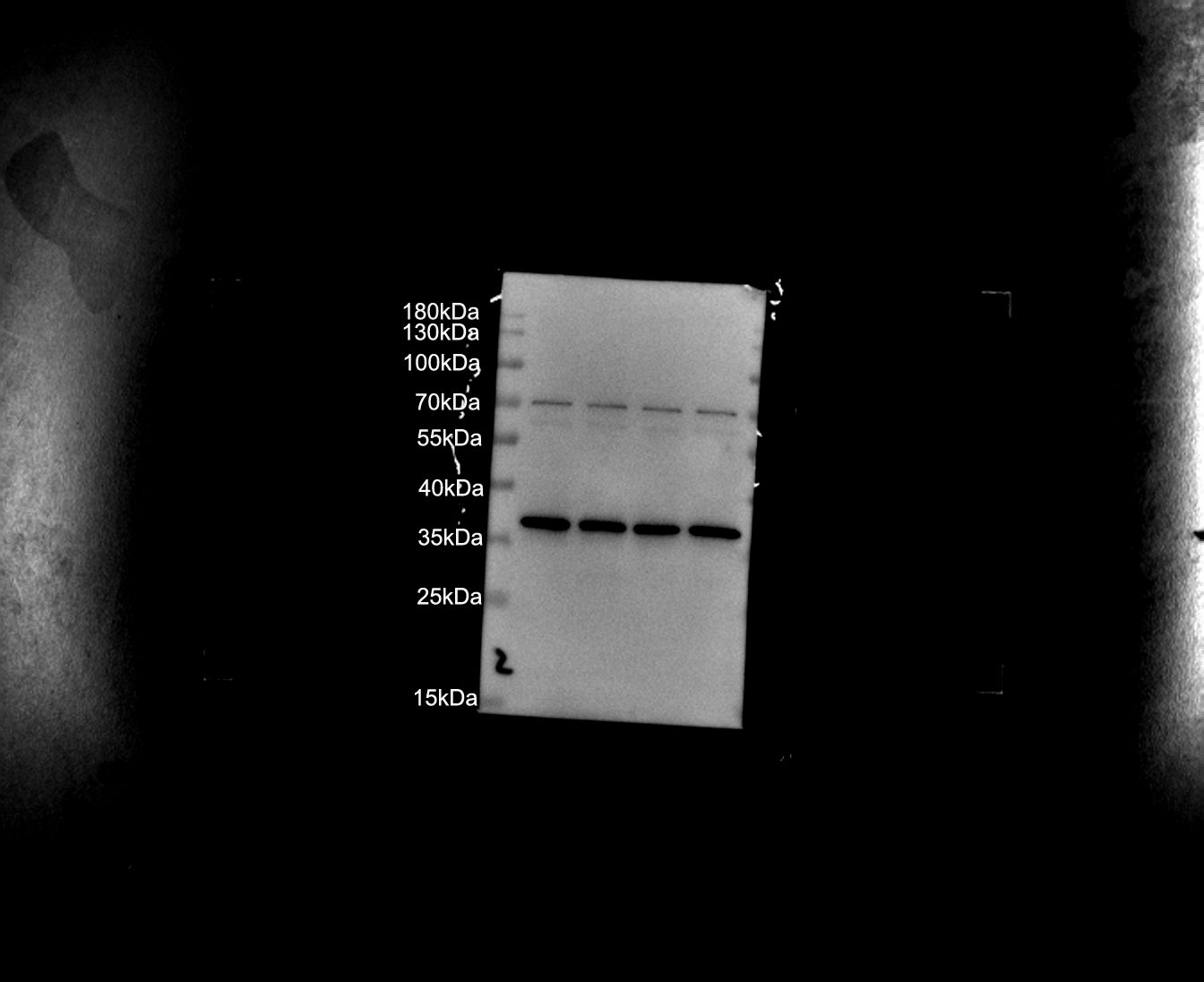


5A-H1299-GRP78


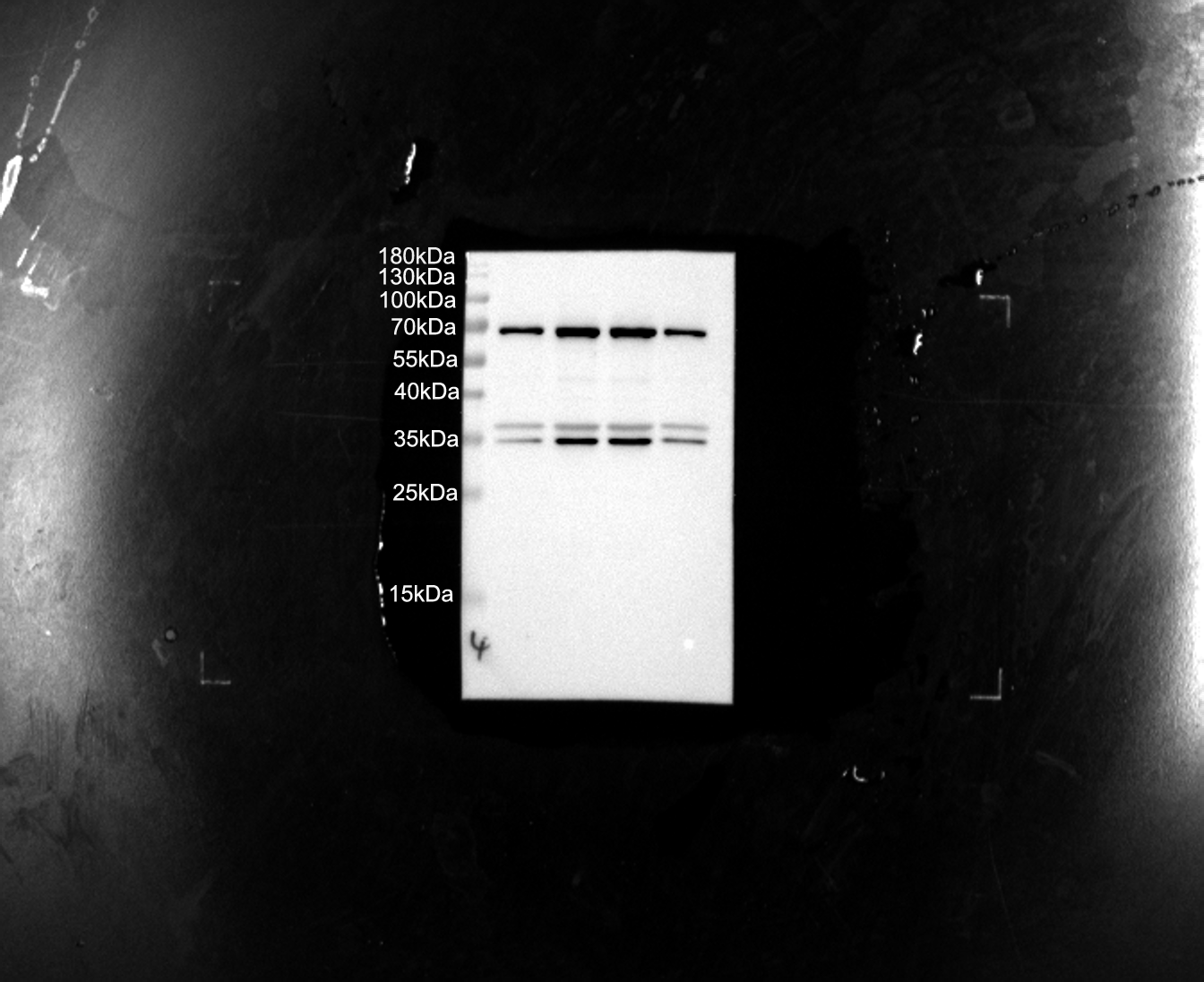


5A-H1299-p-EIF2a


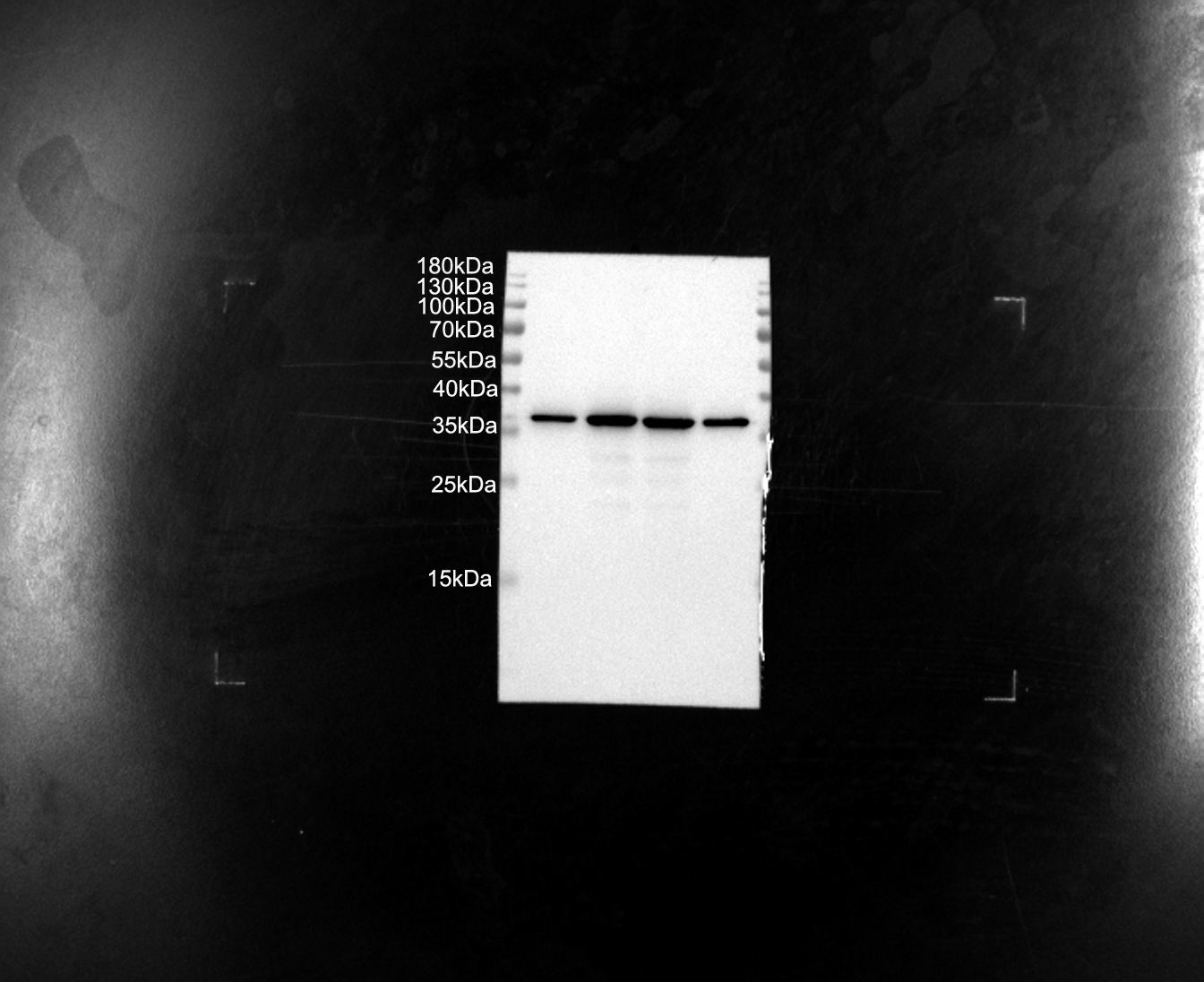


5A-H1299-p-PERK


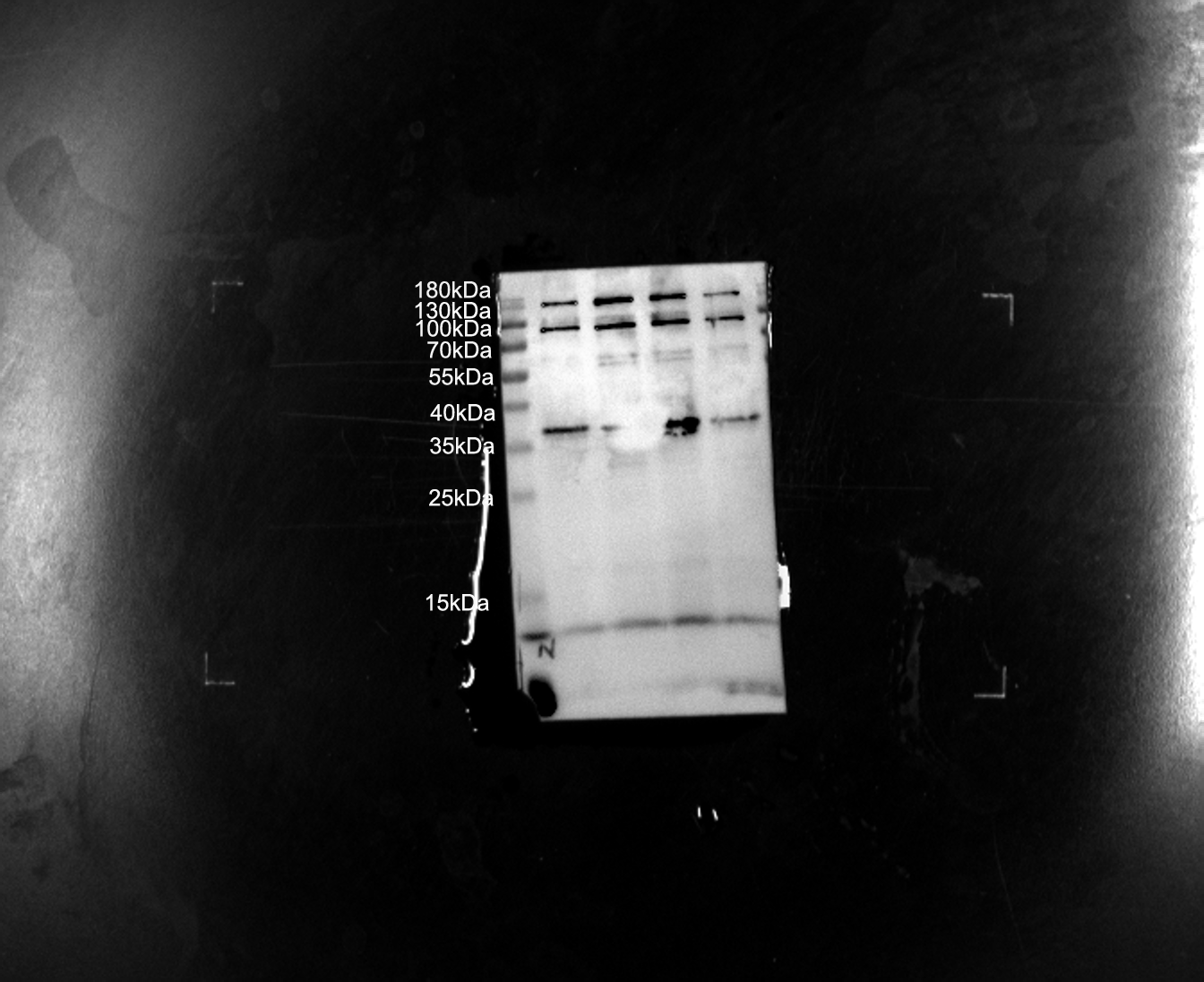


**Figure 5B. ICD-related proteins were examined by western blot.**

5B-A549-CRT


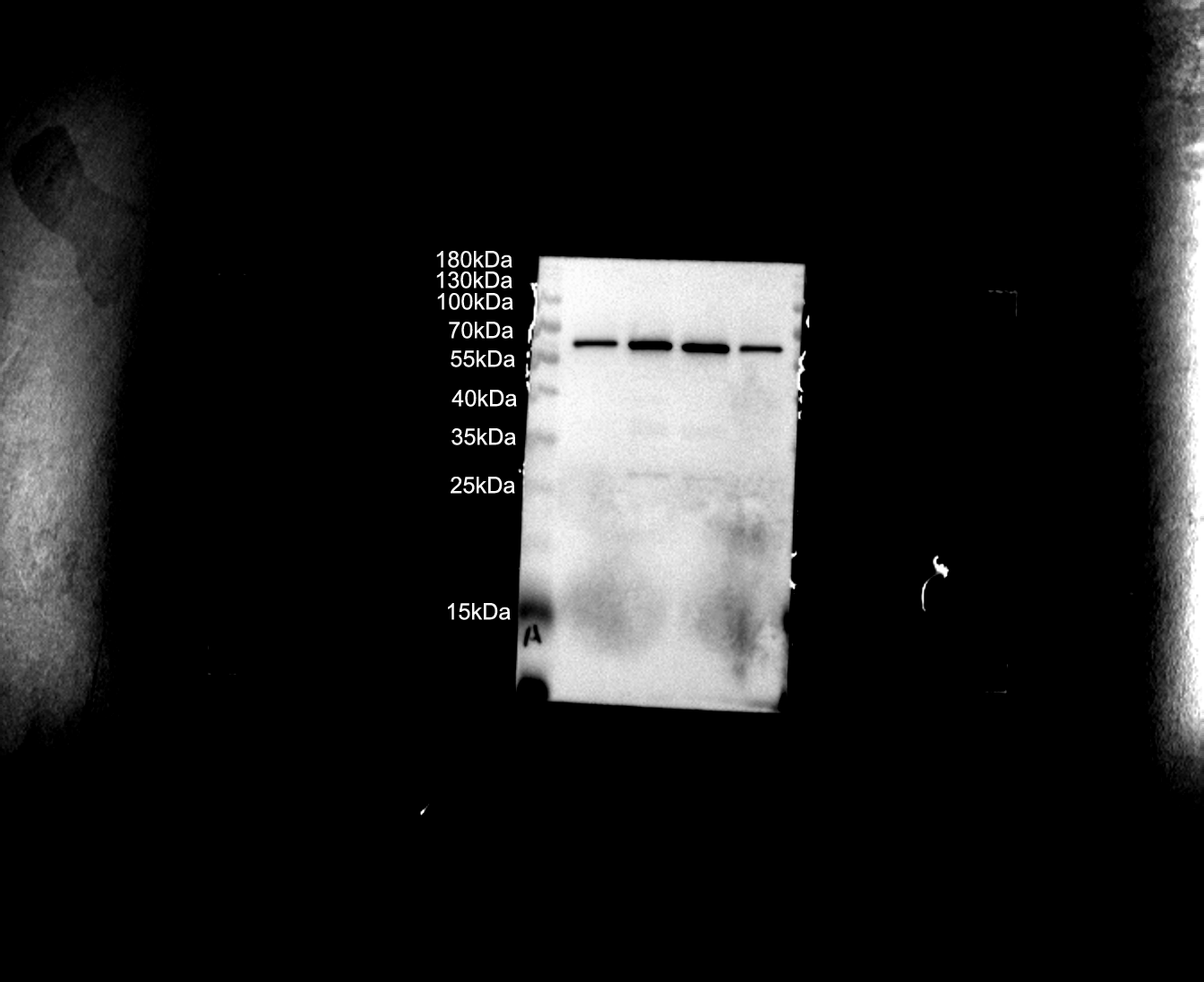


5B-A549-GAPDH


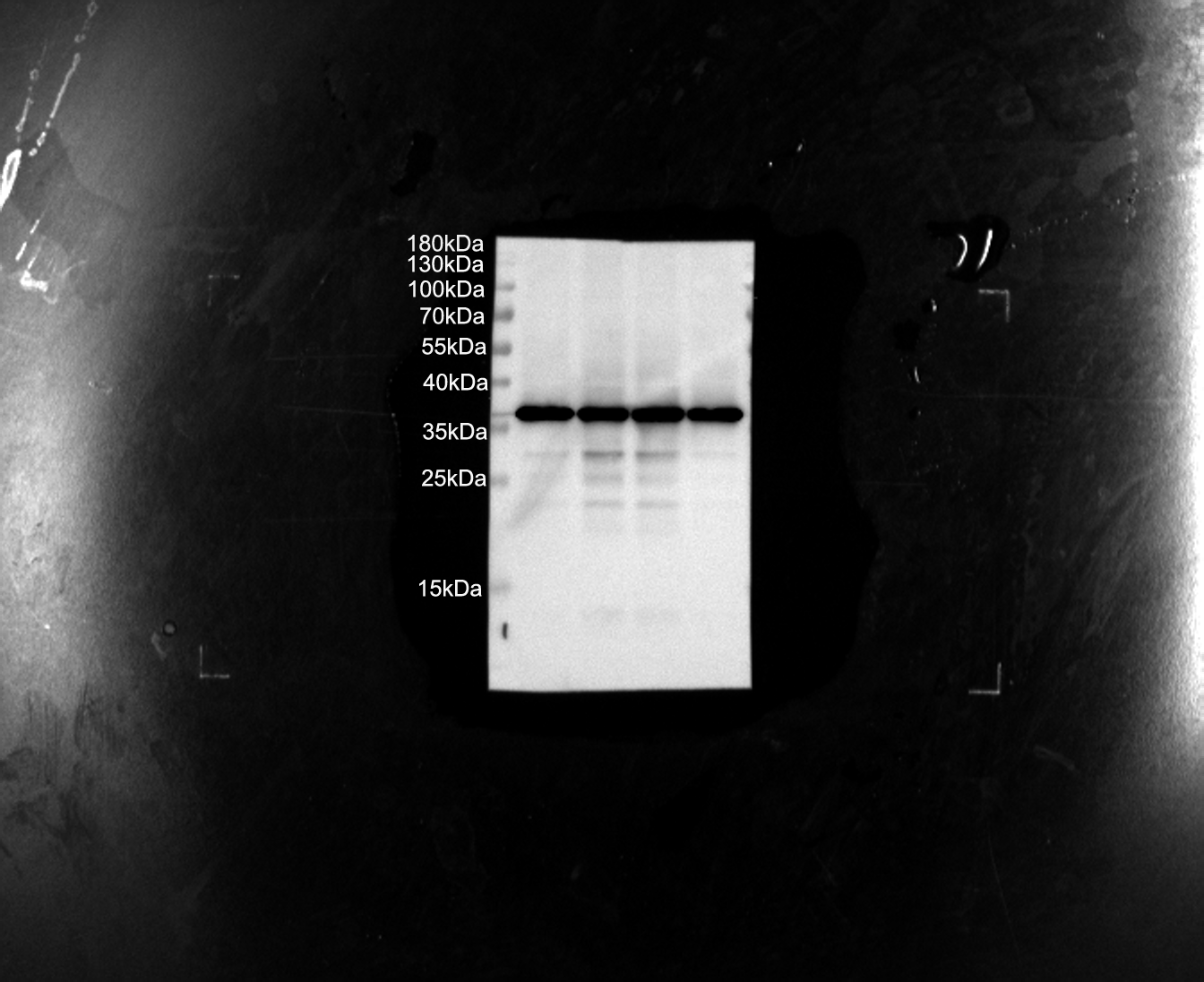


5B-A549-HMGB1


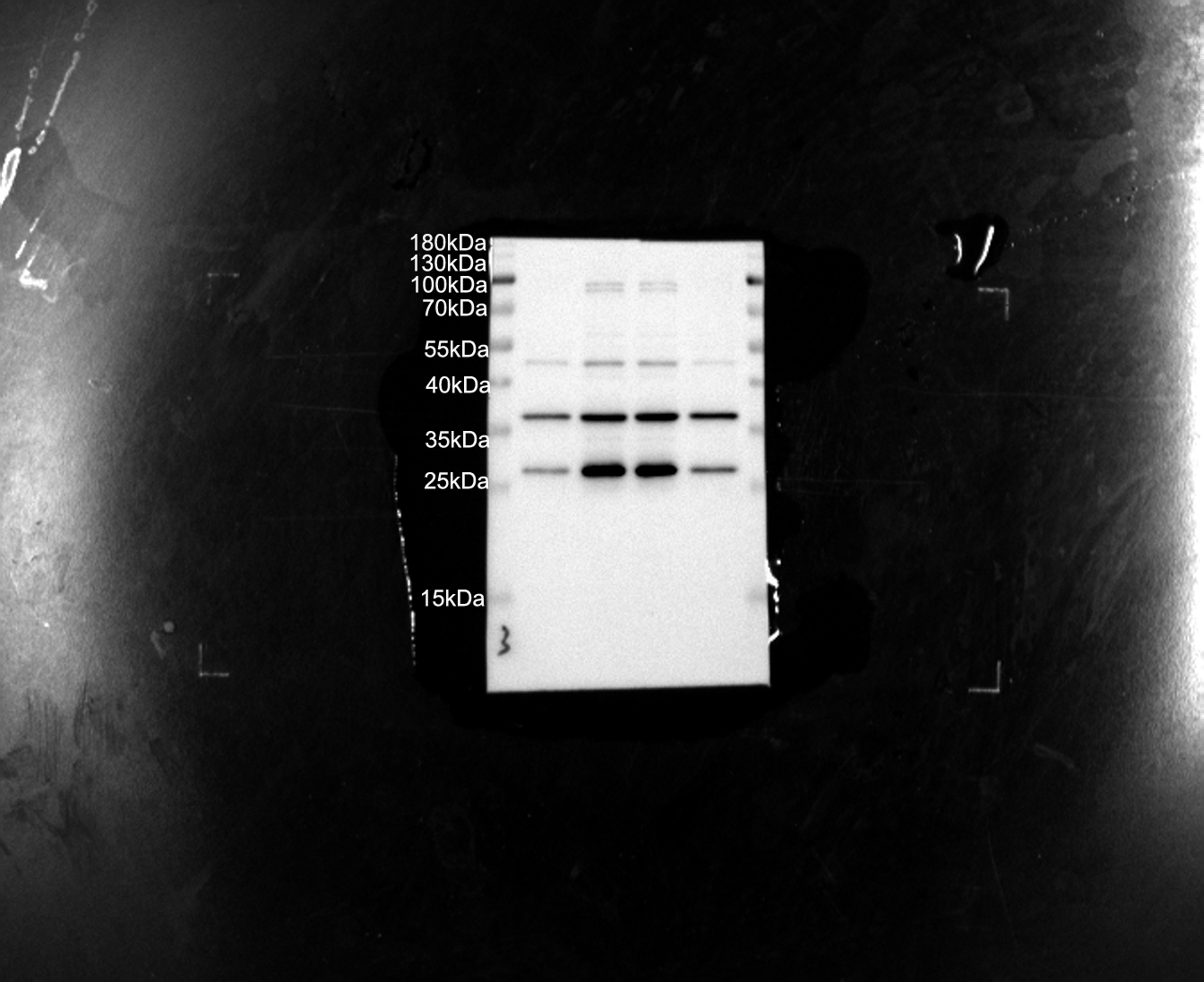


5B-H1299-CRT


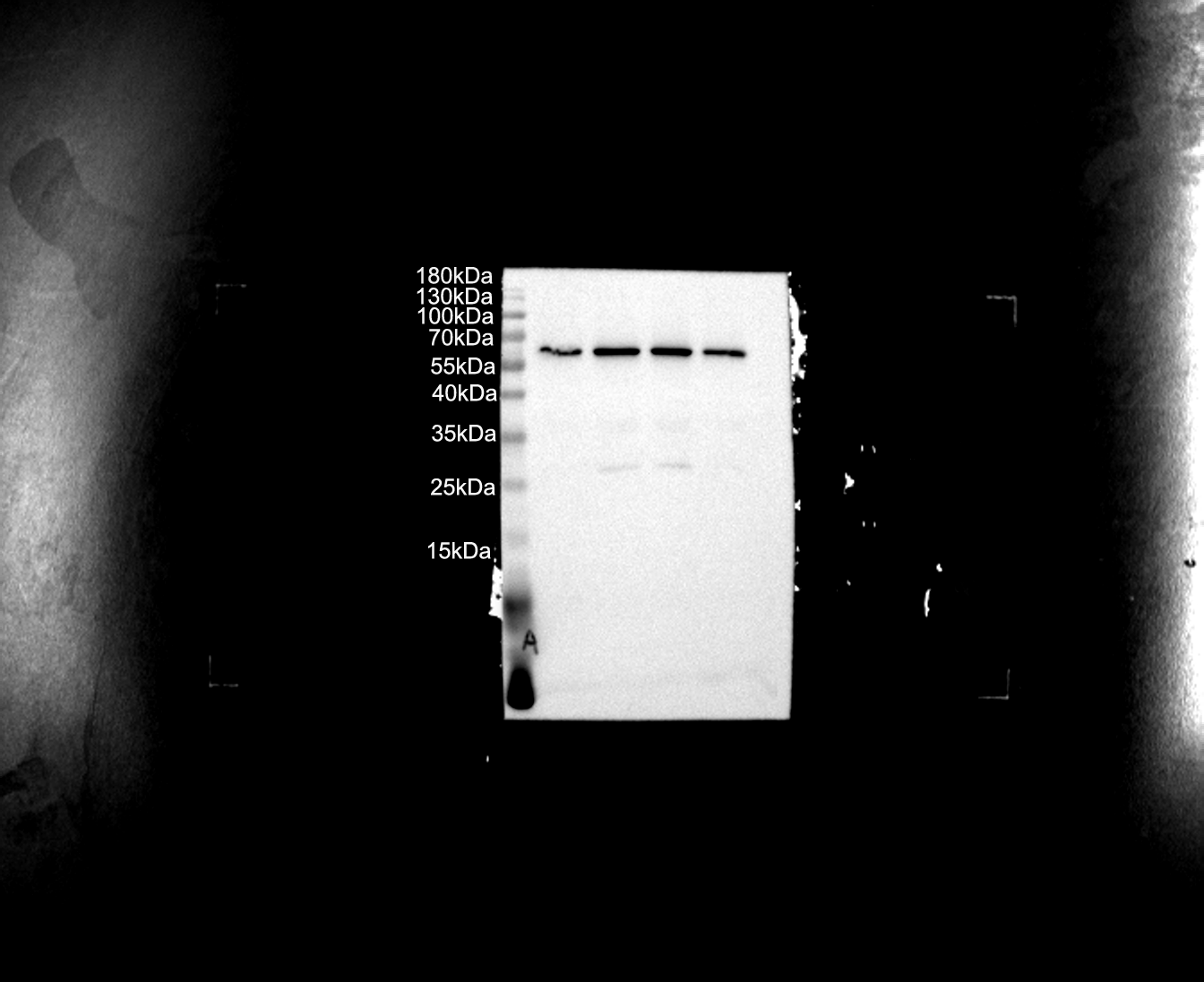


5B-H1299-GAPDH


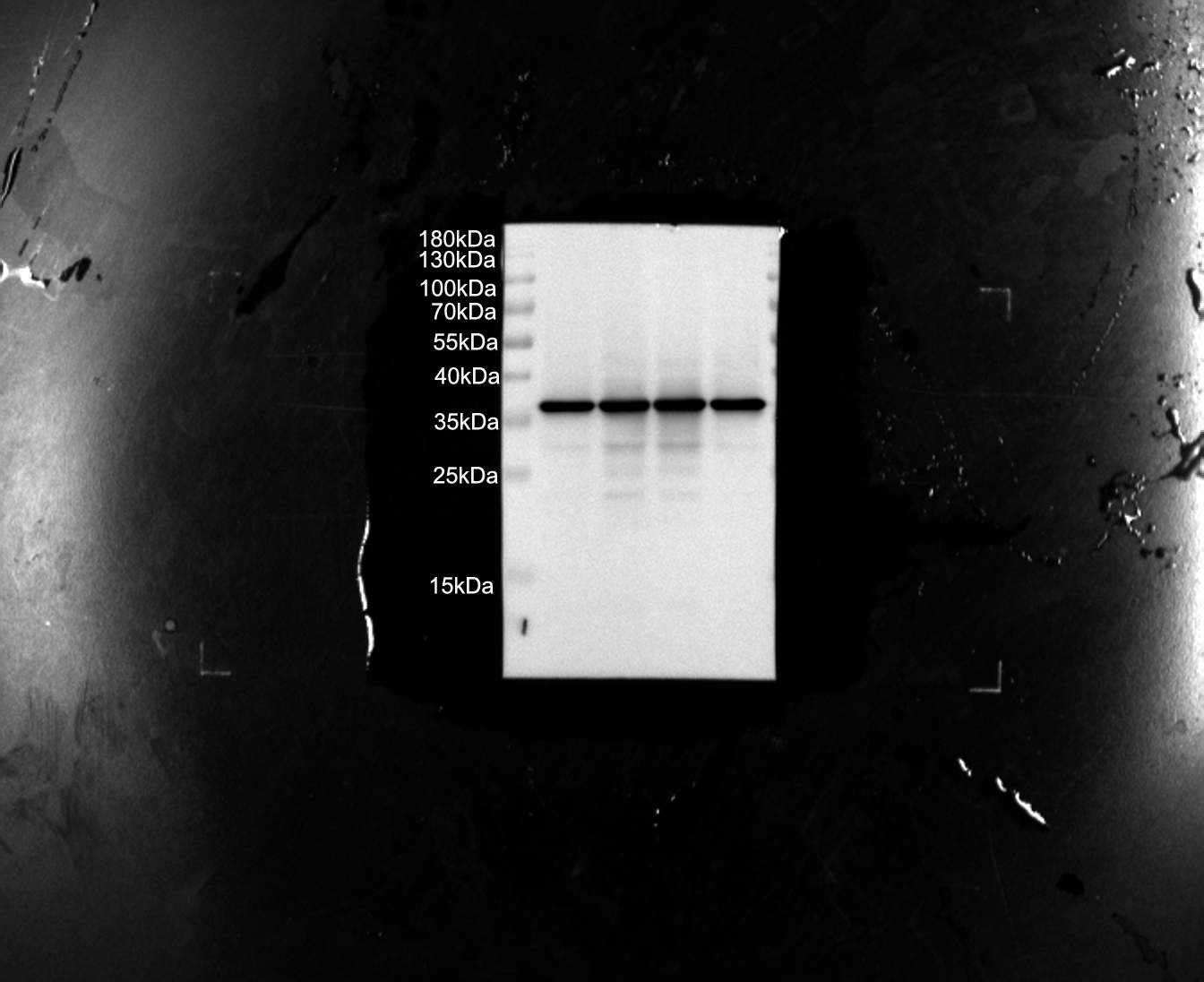


5B-H1299-HMGB1


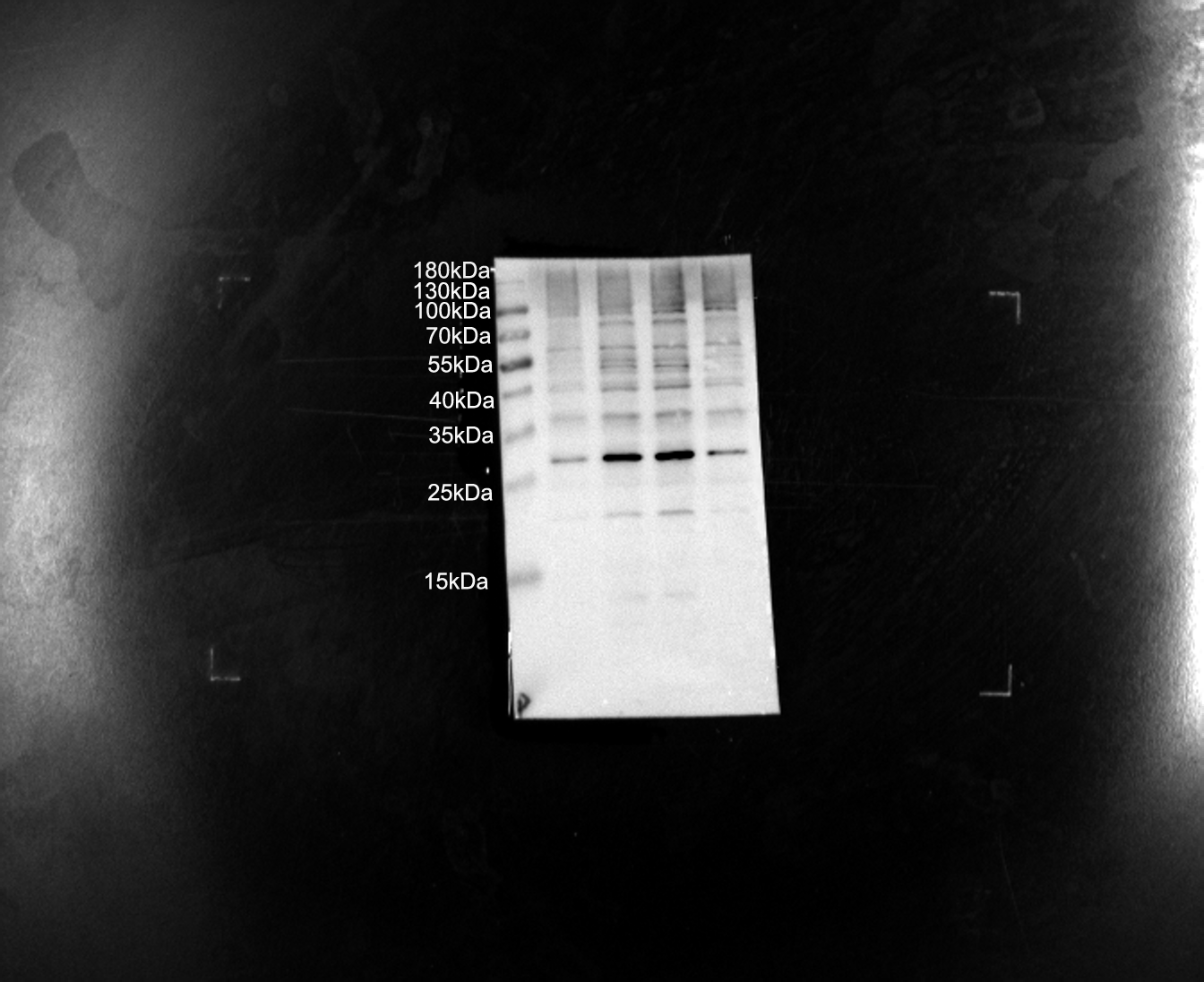


**Figure S1D. ER stress-related proteins were examined by western blot.**

S1D-A549- p-PERK


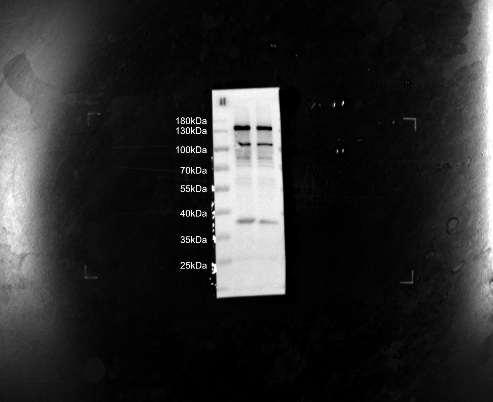


S1D-A549- p-elF-2a


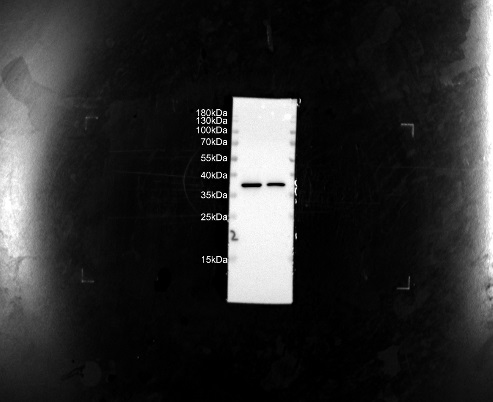


S1D-A549- GRP78


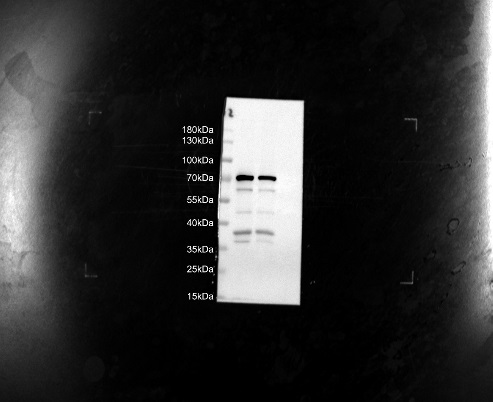


S1D-A549- CHOP


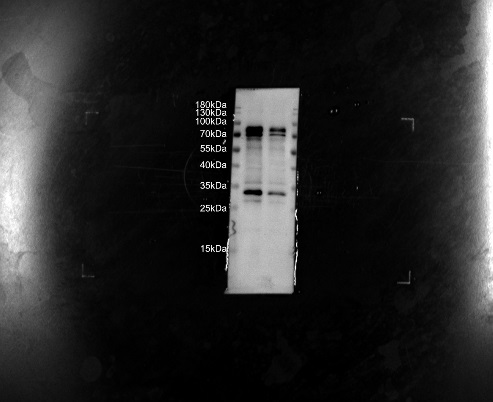


S1D-A549- GAPDH


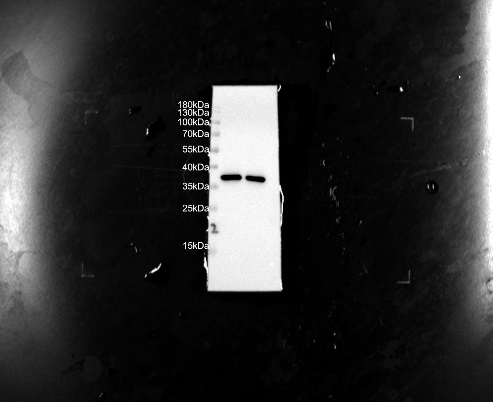


S1D- H1299- p-PERK


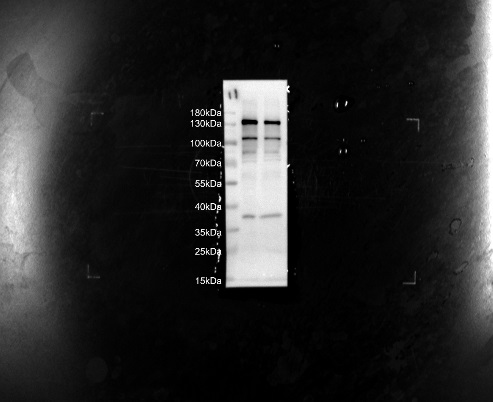


S1D-H1299- p-elF-2a


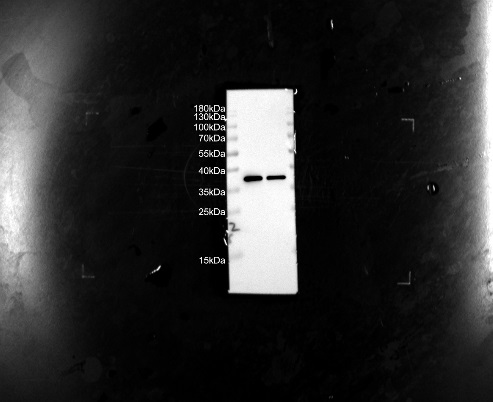


S1D-H1299- GRP78


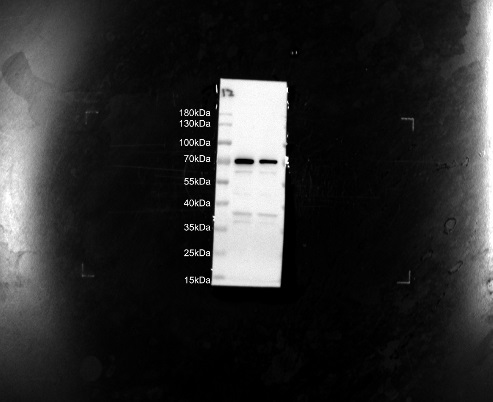


S1D-H1299- CHOP


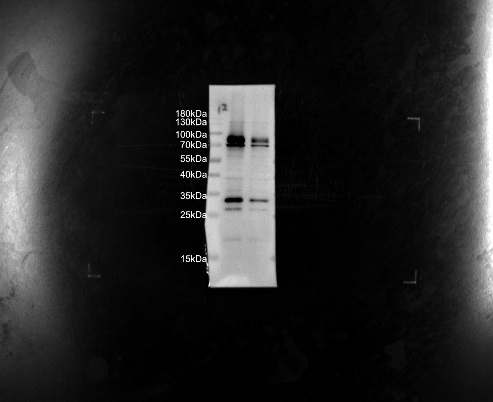


S1D-H1299- GAPDH


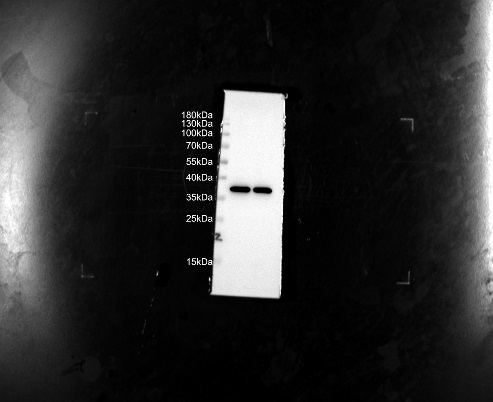

Supplement: Supplementary file 2 — Additional file 2. [file 12906_2023_4221_MOESM2_ESM.docx]
